# The evolution of BRAF-targeted therapies in melanoma: overcoming hurdles and unleashing novel strategies

**DOI:** 10.3389/fonc.2024.1504142

**Published:** 2024-11-08

**Authors:** Saber Imani, Ghazaal Roozitalab, Mahdieh Emadi, Atefeh Moradi, Payam Behzadi, Parham Jabbarzadeh Kaboli

**Affiliations:** ^1^ Shulan International Medical College, Zhejiang Shuren University, Hangzhou, Zhejiang, China; ^2^ Noncommunicable Diseases Research Center, Fasa University of Medical Sciences, Fasa, Iran; ^3^ Department of Biology, Science and Research Branch, Islamic Azad University, Tehran, Iran; ^4^ Department of Life Sciences and System Biology, University of Turin, Turin, Italy; ^5^ Department of Microbiology, Shahr-e-Qods Branch, Islamic Azad University, Tehran, Iran; ^6^ Department of Biochemistry, Faculty of Medicine, Medical University of Warsaw, Warsaw, Poland

**Keywords:** melanoma, BRAF inhibitors, MEK inhibitors, targeted therapy, precision medicine

## Abstract

Melanoma, a highly aggressive form of skin cancer, poses a significant global health burden, with 331,647 new cases and 58,645 deaths reported in 2022. The development of melanoma is influenced by various factors, including sunlight exposure and BRAF^V600^ mutations that activate the MAPK/ERK pathway. The introduction of BRAF and MEK inhibitors has revolutionized the treatment landscape for melanoma patients. However, innate and acquired therapeutic resistance remains a significant challenge. This review provides a comprehensive overview of the current state of BRAF-targeted therapies in melanoma, highlighting the efficacy and limitations of FDA-approved combinations of BRAF and MEK inhibitors such as vemurafenib, dabrafenib, trametinib, and cobimetinib. The review also explores the off-target effects of BRAF inhibitors on endothelial cells, emphasizing the need for more selective therapies to minimize vascular complications and metastatic potential. The article also discusses potential druggable targets, including ERK5, CD73, ALDH1A1, PLA1A, and DMKN, which are promising in addressing diagnostic hurdles and guiding personalized therapeutic decisions. Recent studies on regorafenib, ERK5 signaling, and CD73 inhibition are highlighted as novel strategies to overcome resistance and improve treatment outcomes. The review also delves into the role of advanced therapeutic tools, such as mRNA vaccines and CRISPR-Cas9, in revolutionizing personalized oncology by targeting specific genetic mutations and enhancing immune responses against melanoma. The ongoing synergy between advancing research, targeted interventions, strategic treatment combinations, and cost-effectiveness evaluations offers a promising pathway to elevate patient outcomes in the persistent battle against melanoma significantly.

## 1 Introduction

According to the Global Cancer Observatory, 331,647 new cases and 58,645 deaths were globally reported in 2022 with melanoma, a deadly skin cancer (1). BRAF^V600E^ mutations and exposure to sunlight are considered risk factors for melanoma development. The common BRAF^V600^ mutations in primary melanomas trigger the activation of the Mitogen-Activated Protein Kinase (MAPK)/extracellular Signal-Regulated Kinase (ERK) pathway. BRAF^V600^ mutations can mainly be found in BRAF^V600E^ and BRAF^V600K^ (2). Introducing inhibitors targeting BRAF and MAPK Kinase (MEK), crucial components of this pathway, marked a significant breakthrough in treating this cancer. However, approximately 15-20% of melanomas exhibit innate resistance to this therapy, and patients frequently develop acquired resistance over time (3). Resistance development is a primary concern, limiting the effectiveness of these inhibitors as the initial treatments (4).

Using BRAF and MEK inhibitors (BRAFi/MEKi) has been a cornerstone in treating melanoma, particularly for those with specific genetic mutations like BRAF^V600E^. Drugs such as vemurafenib and dabrafenib have shown significant efficacy in targeting these mutations. Similarly, MEK inhibitors like trametinib and cobimetinib provide alternative or combinatorial therapeutic options for patients with RAS/RAF/MAP pathway-driven cancers. However, a significant limitation of BRAF/MEKi-based therapies is the frequent observation of therapeutic resistance. This resistance can arise due to aberrant pathway activation, metabolic reprogramming, and cancer cells’ genetic and epigenetic landscape alterations. Understanding the specific mutations in the RAS/RAF/MAP pathway is critical for developing effective targeted therapies (5, 6).

Moreover, the off-target effects of BRAF inhibitors on endothelial cells have been found to cause significant vascular complications. These drugs disrupt the MAPK/ERK signaling pathway that regulates cytoskeletal dynamics and cell junction integrity, leading to increased vascular permeability and potential risks such as enhanced metastatic potential of tumor cells and complications related to vascular leakage. These findings underscore the need for monitoring vascular health in patients treated with BRAFi and developing strategies to mitigate these side effects, such as adjunct therapies that protect endothelial integrity or the development of more selective BRAFi that minimize off-target effects (7).

**Figure 1:**
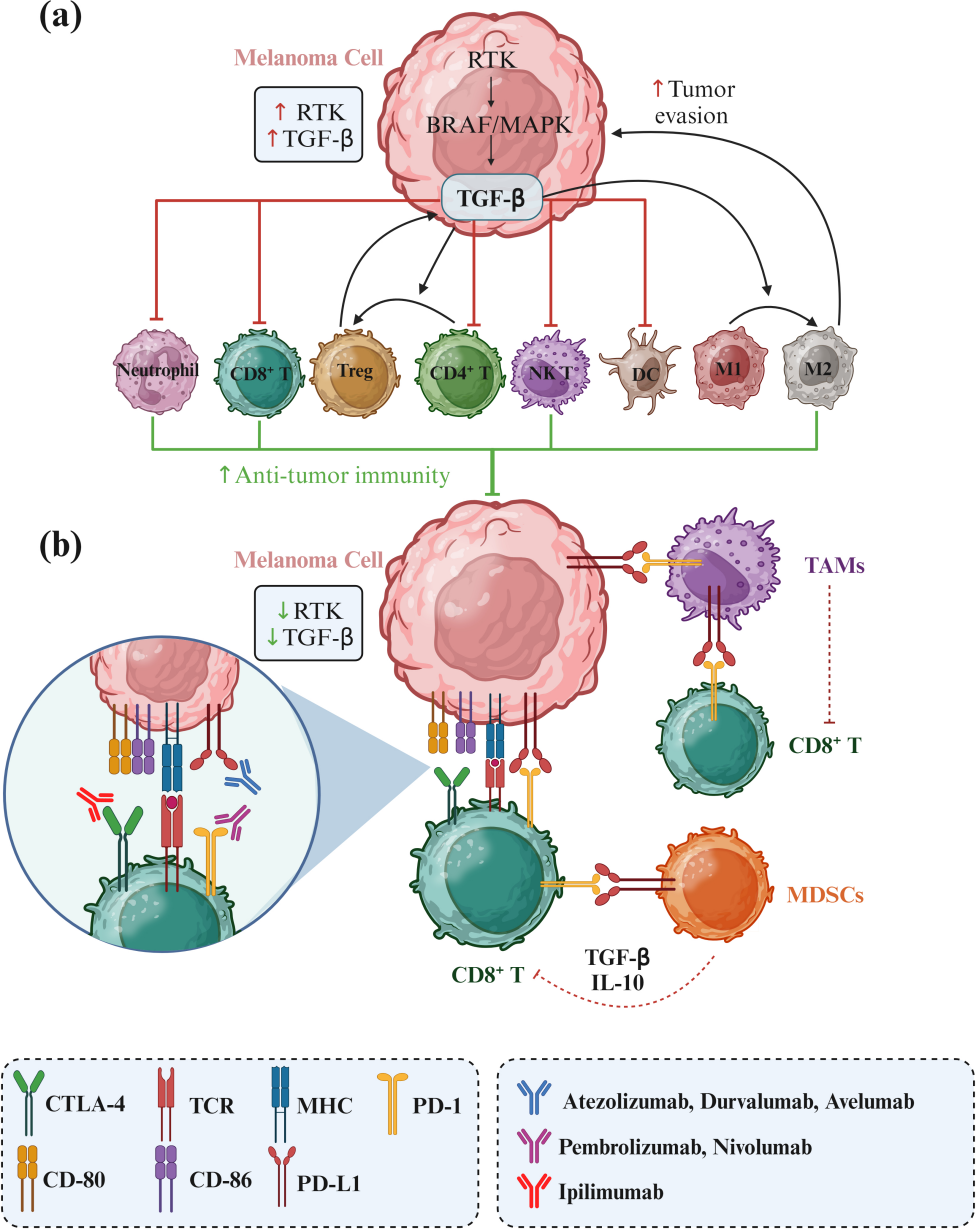
RTK/RAF/MAPK signaling in melanoma and its effects on immune cell activity. Receptor tyrosine kinase (RTK) upregulation enhances the RAF/MAPK pathway, leading to increased TGF-β secretion by melanoma cells. TGF-β promotes the transition of CD4^+^ T cells to regulatory T cells (Tregs) and the polarization of M1 to M2 tumor-associated macrophages (TAMs), facilitating tumor progression and immune evasion. Additionally, immune checkpoints PD-L1 and CD80/CD86 on melanoma cells bind to their counterparts PD-1 and CTLA4, respectively, to suppress immune cell activity against tumor cells. Consequently, immunotherapies targeting these immune checkpoints can significantly improve the treatment of melanoma.

In parallel, PD-1/PD-L1 and CTLA-4 inhibitors (**Figure 1**) have significantly impacted the treatment landscape of melanoma, particularly in advanced stages. These immune checkpoint inhibitors block cancer cells’ proteins to evade an immune response, thereby allowing the immune system to recognize and attack the cancer cells more effectively (8–10). One promising alternative for patients who have failed prior treatments is Tumor-Infiltrating Lymphocyte (TIL) therapy, particularly lifileucel (LN-44). Lifileucel is currently under review by the FDA for approval and involves expanding patient-specific TILs, primarily CD8^+^ and CD4^+^ T cells with an effector memory phenotype (11). This therapy has shown long-lasting and profound responses, suggesting its potential as a therapeutic option for advanced melanoma patients with high tumor burden. However, while these results are promising, further research is necessary to fully understand the potential and long-term benefits of lifileucel therapy.

Regorafenib, a multitargeted kinase inhibitor, has shown promise in advanced melanoma patients who had previously progressed on anti-PD-1, anti-CTLA-4, and BRAF/MEK inhibitors, 42.8% of BRAF^V600^ mutation-positive patients treated with regorafenib combined with BRAFi/MEKi showed a partial response, including regression of brain metastases. This suggests that regorafenib, especially when combined with other targeted therapies, may provide benefits for advanced melanoma patients who have exhausted other treatments (12).

While PD-1/PD-L1 and CTLA-4 inhibitors have revolutionized melanoma treatment, challenges such as resistance necessitate exploring alternative and combinatorial therapies. Lifileucel TIL therapy (11, 13), regorafenib (12), ERK5 inhibitors (14, 15), and targeting CD73 (16) represent other promising avenues to enhance treatment efficacy and manage resistance in advanced melanoma.

Our review manuscript uniquely contributes to the field by offering a comprehensive analysis transcending the traditional focus on BRAF-targeted therapy alone. While existing reviews concentrate on specific aspects of BRAF-targeted treatment, we provide a holistic perspective that translates findings from *in vitro* studies to the clinical area, encompassing FDA-approved drugs and their real-world implications. Moreover, our review goes beyond the conventional scope by integrating the discussion of immunotherapy with BRAF-targeted therapy, exploring the synergies between these treatment modalities and their potential impact on patient outcomes. By incorporating discussions on novel developments and emerging biomarkers, we offer a forward-looking perspective highlighting melanoma therapy’s evolving landscape and the potential for more tailored and effective treatment approaches. Overall, our review is distinguished by its comprehensive approach that bridges the gap between preclinical research, clinical application, FDA-approved therapies, immunotherapy integration, and innovative markers in the context of BRAF-targeted treatment for melanoma.

## 2 RAF/MEK signaling

ARAF, BRAF, and CRAF are part of a triad of protein-serine/threonine kinases that hold pivotal positions within the RAS-RAF-MEK-ERK signaling pathway. This intricate cascade orchestrates many fundamental cellular processes, encompassing apoptosis, cell cycle progression, differentiation, proliferation, and the transformation of cells into a malignant state. Several decades ago, the primary mammalian MAPK, denominated ERK, was discovered (17–19).

The MAPK/ERK pathway is triggered by upstream genomic events or the activation of many signaling pathways that converge at this crucial junction. This pathway maintains strict regulation under normal circumstances through the actions of phosphatases and bidirectional communication with other pathways, including the protein kinase B/mammalian target of the rapamycin (AKT/mTOR) pathway. Recent findings suggest that the MAPK/ERK signaling hub can serve as both a tumor suppressor and a more typical pro-oncogenic signal, with the dominant effect contingent on signal intensity and the specific tissue or context in which the signal becomes aberrantly activated (20).

Notably, RAS mutations are prevalent in 15-30% of all human cancers, while BRAF mutations are found in 30-60% of melanomas, 30-50% of thyroid cancers, and 5-20% of colorectal cancers. The activation of RAF kinases necessitates a series of events, including their interaction with RAS-GTP, followed by dephosphorylation/phosphorylation facilitated by SRC family protein-tyrosine kinases and other protein-serine/threonine kinases. Furthermore, forming specific RAF dimers is crucial for achieving total kinase activity (21). A significant turning point occurred when the v-RAF oncogene was found to induce the constitutive activation of MAPK and MKK in NIH-3T3 cells, highlighting RAF as a direct regulator of MKK. This discovery further solidified RAF’s status within the MEK kinase (MKKK) family (22).

**Figure 2:**
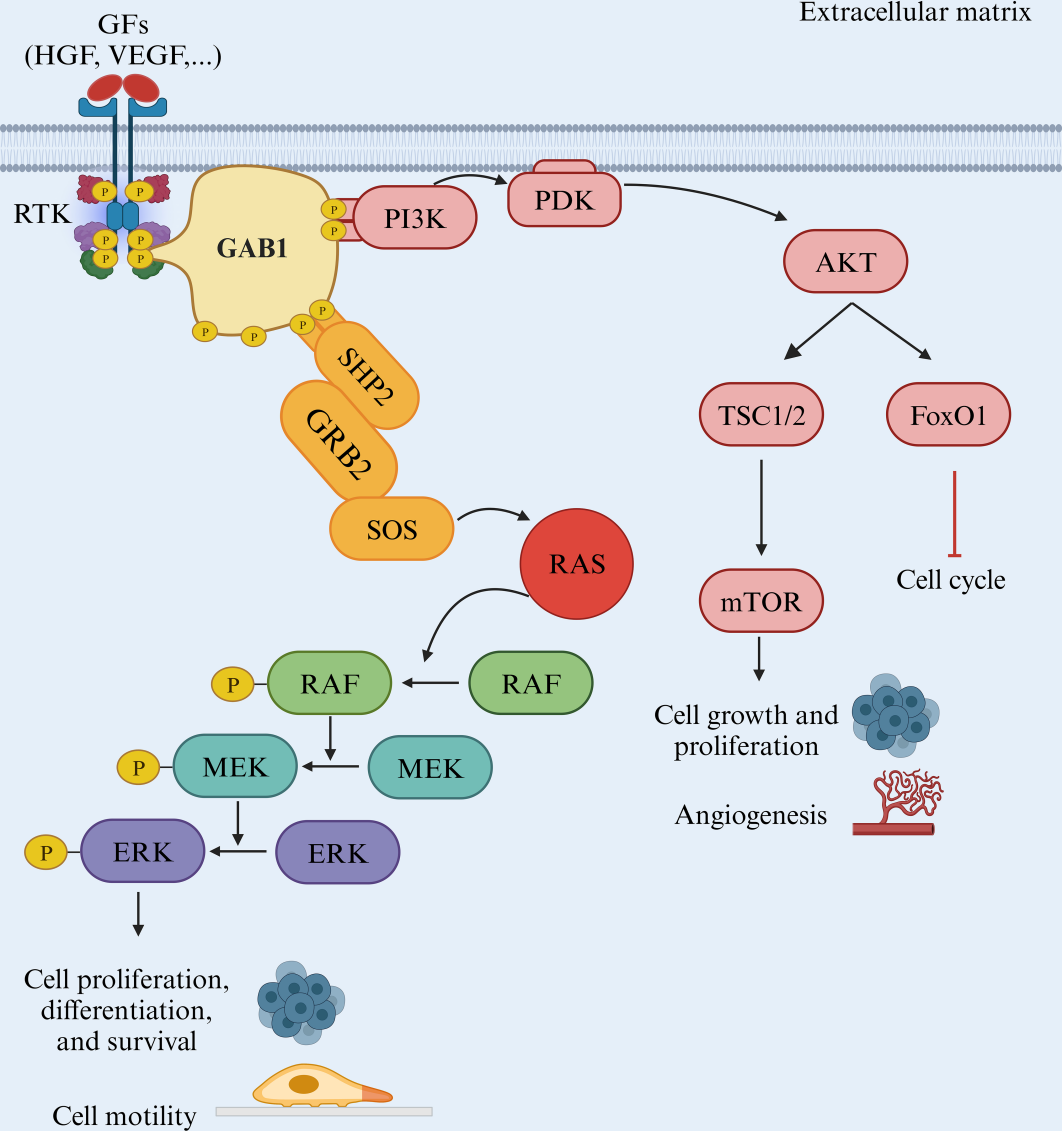
The BRAF/MAPK pathway. The RAF/MEK/ERK pathway is a critical signaling pathway in the cell that plays a pivotal role in various cellular processes, including cell proliferation and metastasis. This pathway is often initiated by receptor tyrosine kinases (RTKs) and involves several key proteins, such as GAB1, GRB2, RAF, MEK, and ERK.

Another vital component of the MAPK pathway is MEK1/2, which undergoes direct activation through phosphorylation of serine residues. These kinases, in turn, phosphorylate ERK1 and ERK2 at threonine and tyrosine residues. ERK1/2, the ultimate kinases in the MAPK signaling cascade, significantly affects cell proliferation, differentiation, and survival. Mutations affecting components of the MAPK pathway are common in various cancer types, making the inhibition of BRAF and MEK, and consequently the suppression of downstream signaling, a promising therapeutic strategy. However, unlike MEK, mutations in ERK are infrequent, with occurrence rates of 8% in cervical cancers and 1.5% in head and neck squamous cell carcinomas (**Figure 2**) (23).

Furthermore, activation of ERK relies on the phosphorylation of specific threonine and tyrosine residues, with dephosphorylation mediated by phosphatases countering this process (18). This led to the postulation that an upstream kinase was responsible for ERK phosphorylation. Indeed, a dual-specificity kinase was discovered, subsequently referred to as MAP kinase activator, commonly known as MKK or MEK. Intriguingly, like MAPK, MKK is subject to negative regulation by phosphatases, implying the presence of an upstream regulatory kinase for MKK (24).

Numerous negative feedback mechanisms tightly control receptor tyrosine kinase (RTK(-mediated MAPK responses, reflecting their central role in the core processes of the network. Two crucial upstream regulators of MAPK (SOS and RAF) are also direct substrates of MAPK. The direct phosphorylation of SOS by MAPK disrupts SOS/growth factor receptor-bound protein 2 (GRB2) interactions, diminishing SOS recruitment to the membrane and reducing RAS activation (25). Additionally, MAPK phosphorylates RAF, its upstream regulator, reducing RAF kinase activity and decreasing phosphorylation of MEK and MAPK. MAPK also phosphorylates docking proteins, creating an additional negative feedback mode. MAPK activation results in the phosphorylation of GRB2-Associated Binding Protein 1 (GAB1), reducing its ability to recruit and activate Phosphoinositide 3-Kinase (PI3K) (26, 27).

The well-established RAF-MEK-ERK signaling cascade, a prominent segment of the MAPK pathway, has significant control over cell proliferation and survival. This cascade kicks into gear upon activating RTK (e.g., VEGFR2) and RAS (e.g., NRAS), setting off a chain of events (28). Changes in this pathway represent some of the most prevalent genetic changes observed in human cancers, with numerous frequently occurring mutations such as BRAF^V600E^. These oncogenic mutations often disrupt normal regulatory mechanisms, fostering uncontrolled cell growth and tumor formation (29). Furthermore, interactions between the RAF-MEK-ERK pathway and other signaling pathways significantly amplify its role in promoting cancer growth (30).

## 3 BRAF vs. MEK – structural features

### 3.1 RAF and MEK structures

**Figure 3:**
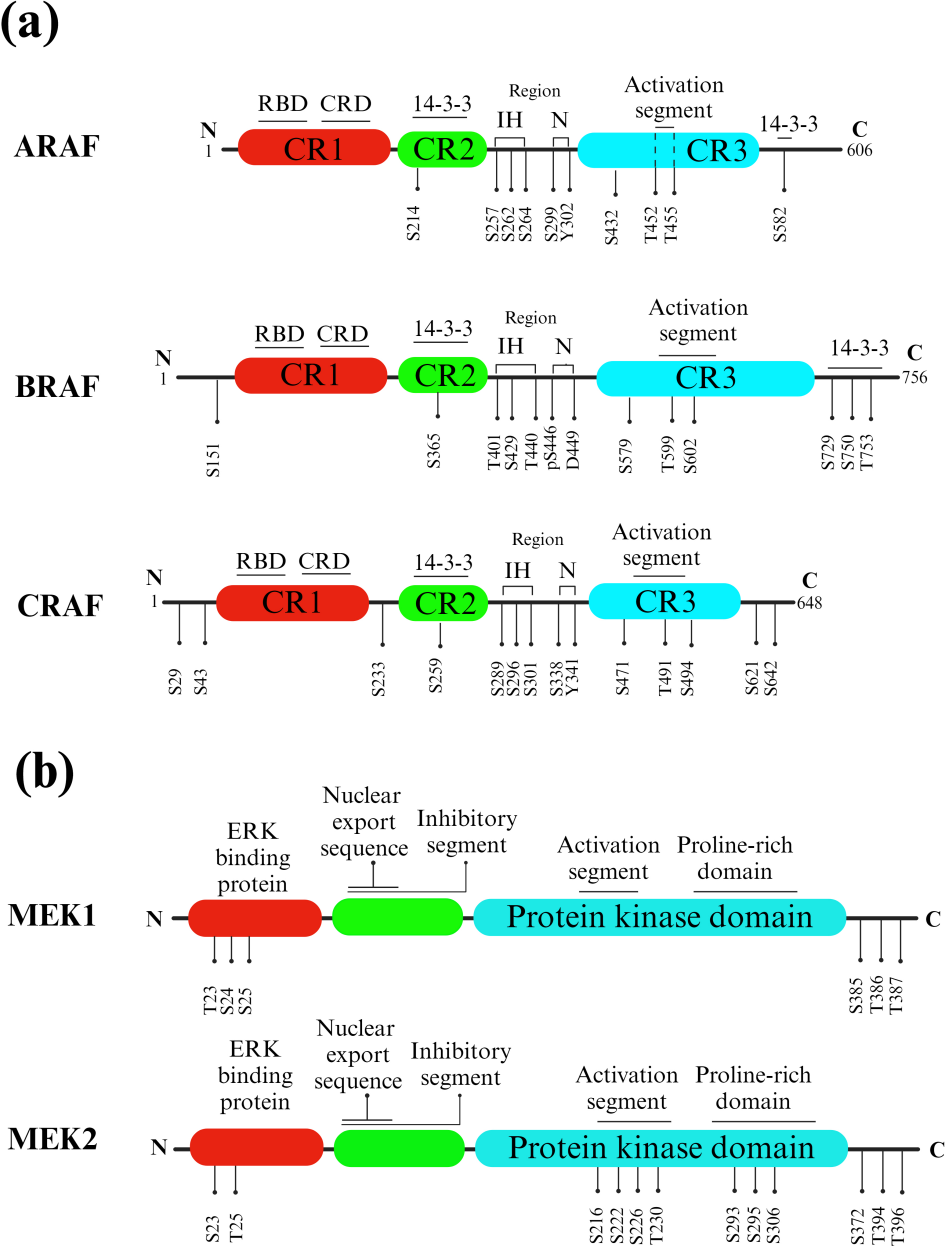
RAF and MEK isoforms and phosphorylation sites. **(A)** RAFs. Key structural features of different RAF isotypes. The three highly conserved regions (CR1, CR2, and CR3) are indicated, with CR1 containing the RAS binding domain (RBD) and cysteine-rich domain (CRD), CR2 comprising serine and threonine regulatory phosphorylation sites, and CR3 housing the P-loop or glycine-rich loop and the kinase domain, including the activation segment. Phosphorylated CR2 (pS365) and the C-terminal region (pS729) of BRAF, as well as phosphorylated CR2 (pS259) and the C-terminal region (pS621) of CRAF, act as binding sites for 14-3-3 proteins. Instead, KRAS is linked to the RAS-binding domain (RBD). **(B)** MEKs. Activated RAF phosphorylates and activates MEK, which is a dual-specificity kinase. MEK has two kinase domains, and it phosphorylates a specific tyrosine and threonine residue on ERK proteins.

RAF proteins are crucial components of the RAS/RAF/MEK/ERK signaling pathway, responsible for transmitting signals from RAS-activated proteins through MEK and ERK kinases. In mammals, there are three closely related RAF genes: ARAF, BRAF, and CRAF (**Figure 3A**). These RAF proteins have three highly conserved regions: CR1, which includes the RAS binding domain and a cysteine-rich domain; CR2, which contains regulatory phosphorylation sites for serine and threonine; and CR3, which features the P-loop or glycine-rich loop and the kinase domain, including the activation segment.

A meaningful connection exists between BRAF and CRAF, which is capable of initiating a BRAF–CRAF–MEK–ERK signaling cascade in both cancerous and normal cells. As a result, BRAF can activate MEK directly or indirectly through the activation of CRAF via heterodimerization (**Figure 3B**) (31). The protein kinase domain of RAF exhibits the typical structure observed in all protein kinases, consisting of a small N-terminal lobe and a larger C-terminal lobe. The small lobe primarily features an antiparallel β-sheet structure and serves to anchor and position ATP. A glycine-rich ATP-phosphate-binding loop, often called the P-loop, is located in the N-terminal lobe (6).

Protein kinases possess two movable lobes that can be separated or come together to open or close the cleft. ATP can access the active site in the open configuration, while ADP can be released. Conversely, the closed conformation aligns specific residues into a catalytically active state. Each lobe has a polypeptide segment that assumes either active or inactive conformations. In the small lobe, this segment corresponds to the major α-helix, the αC-helix. The αC-helix undergoes rotational and translational movements with the rest of the lobe. This dynamic behavior influences the activation or deactivation of a portion of the active site. As mentioned, two RAF subunits combine to form side-to-side dimers involving the regulatory αC helices. In the large lobe, the activation segment adapts to affect the ATP-binding site’s accessibility. The activation segment in all protein kinases typically commences with a DFG (Asp/Phe/Gly) amino acid sequence. In the inactive state, the phenylalanine side chain occupies the ATP-binding pocket, and the aspartate side chain faces away from the active site, a configuration referred to as the DFG Asp-out conformation. In contrast, the active state involves the rotation of the phenylalanine side chain out of the ATP-binding pocket, with the aspartate side chain directing into the ATP-binding pocket and coordinating with Mg^2+^. This arrangement is termed the DFG-Asp in conformation (6).

Most protein kinases possess activation segments that contain one or more phosphorylation sites. Enzymes typically phosphorylate these sites belonging to the same protein kinase family, although other protein kinases may also perform this function. For example, the RAF kinases catalyze the phosphorylation of two serine residues in the activation segment, activating MEK1/2. In many protein kinases, a gatekeeper residue separates the adenine-binding site from an adjoining hydrophobic pocket. Specific kinase inhibitors bind to the adenine-binding site and extend into this hydrophobic pocket. Specific kinase inhibitors target this site. The mutation of a gatekeeper residue (threonine) to a larger one (methionine) can prevent the binding of kinase inhibitory drugs. This mechanism is one way to develop resistance to drugs in clinical settings and can be employed experimentally to generate enzymes that do not interact with a specific drug (32).

Subdomains are characterized by conserved amino acid residue patterns constituting protein kinases’ catalytic core. Among these, three amino acids forming a K/D/D (Lys/Asp/Asp) motif exemplify BRAF’s catalytic properties. K578 in BRAF establishes salt bridges with ATP’s γ-phosphate. D576, a catalytic loop base, positions the substrate protein’s serine or threonine group and extracts a proton from the –OH group, thereby facilitating the nucleophilic attack of oxygen on the γ-phosphorus atom of Mg^2+^-ATP (33). In addition, K578 is the primary site for ubiquitin attachment during EGF-induced K63-linked polyubiquitination of BRAF, and this process plays a critical role in the activation of ERK driven by EGF (34).

Furthermore, D594 marks the activation segment’s initial residue. In most protein kinases, the activation segment begins with DFG and ends with APE (Ala/Pro/Glu), while in ARAF, it ends with AAE (Ala/Ala/Glu). D594 binds to Mg^2+^, which coordinates ATP’s β- and γ-phosphates. On the other hand, the large lobe is predominantly composed of α-helical structures. It is responsible for binding to its protein substrate, MEK1/2 (21).

### 3.2 RAF and MEK structural biology

The process of loading GTP onto RAS results in the activation of RAF, which subsequently triggers the activation of MEK by phosphorylating two serine residues in its activation loop (A-loop), precisely, S218 and S222 (35). Notably, helix C in both MEK and BRAF remains inactive and outward, and this state is stabilized by the A-loop helix in MEK and the inhibitory turn in BRAF. In this complex, specific residues, including BRAF^N660^, BRAF^N661^, and BRAF^R662^, along with the MEK activation loop, create a binding pocket (36).

A study aimed to investigate how mammalian 14-3-3 proteins activate RAF kinases. It was observed that BRAF had a more diverse association with 14-3-3 proteins *in vivo* compared to ARAF and CRAF. *In vitro* tests also indicated that ARAF had lower affinities for specific 14-3-3 isoforms. This suggested that 14-3-3 proteins interacted selectively with RAF isoforms. Homodimeric and heterodimeric forms of 14-3-3 participate in RAF activation. Furthermore, the research revealed that the activities of RAF isoforms were differentially regulated by their C-terminal and internal 14-3-3 binding domains. The study also observed that prohibitin, a scaffold protein, interfered with the internal 14-3-3 binding site in CRAF (37).

**Figure 4:**
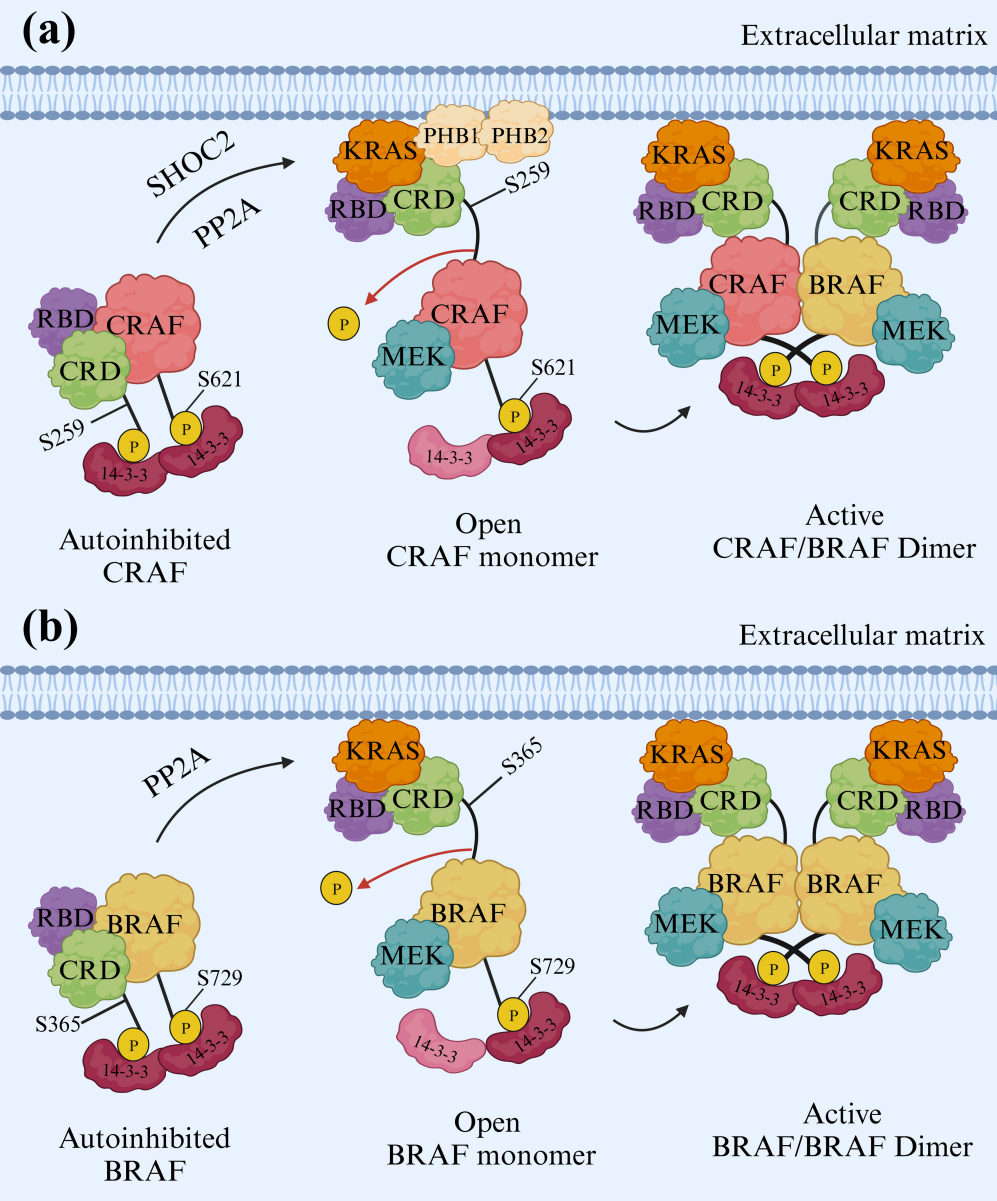
Activation mechanisms of CRAF and BRAF. **(A)** CRAF Activation. To activate CRAF, the SHOC2 complex removes phosphate groups from S259 of CRAF. This dephosphorylation is vital, enabling CRAF to pair with other RAF kinases when stimulated by growth factors. The formation of these RAF heterodimers and removing the inhibitory “S259” site are crucial for CRAF activation. Prohibitin 1 (PHB1) also plays a key role by directly interacting with CRAF and stimulating ERK1/2. **(B)** BRAF Activation. BRAF activation resembles a lock-and-key mechanism. BRAF forms a complex with its partner, MEK, and a 14-3-3 dimer in its inactive state. The 14-3-3 dimer acts like a lock, encircling specific sites (pS365 and pS729) on both sides of the BRAF kinase domain. This interaction effectively keeps the BRAF inactive by preventing dimerization, a crucial step for activation. Two types of 14-3-3 proteins, single and mixed types, actively participate in RAF activation. In this arrangement, the cysteine-rich domain (CRD) is centrally shielded from interactions with the cell membrane and RAS, while the RAS-binding domain (RBD) of BRAF is exposed and ready to interact with RAS.

Furthermore, research has shown that the resurgence of MAPK signaling, achieved through CRAF overexpression and irregularity, is a mechanism for developing resistance to vemurafenib in melanoma. Prohibitins (PHBs) are highly conserved proteins that regulate the cell cycle, senescence, and tumor suppression (38). Prohibitins play a role in governing the activation of CRAF kinase, which connects to RAS in a GTP-dependent manner, thus triggering the MAPK pathway. Prohibitin 1 (PHB1) is a crucial component for CRAF-mediated activation of ERK1/2 via direct binding to CRAF. PHB1 forms stable heteromers with Prohibitin 2 (PHB2). Phosphorylation of PHB1 at Thr258, facilitated by AKT1, increases CRAF association, resulting in the hyperactivation of ERK1/2 kinases, promoting the metastasis of cervical cancer cells to lymph nodes (39, 40). On the other hand, SHOC2 complex-mediated dephosphorylation of S259 CRAF is crucial for growth factor-induced RAF heterodimerization and MEK dissociation from BRAF. In addition, there are SHOC2-independent mechanisms for activating the RAFs and ERK pathways, relying on the N-region phosphorylation of CRAF. While heterodimerization of RAF kinases and removing an inhibitory site marked “S259” are important steps for CRAF activation, the precise mechanisms and dynamics remain unclear. A ternary complex composed of SHOC2, KRAS, and PP1, known as the SHOC2 complex, serves as a CRAF S259 holophosphatase (**Figure 4A**) (41).

Additionally, BRAF remains in an inactive state when forming a complex with its substrate MEK and a 14-3-3 dimer. In this autoinhibited arrangement, the 14-3-3 dimer attaches to serine phosphorylation sites situated on either side of the BRAF kinase domain (pS365 and pS729). This interaction confines both the BRAF kinase and cysteine-rich domains (CRD) within a protective structure, preventing BRAF dimerization, which is essential for its activation (42). The BRAF/MEK1 complex is considered to exist in an autoinhibited state, but the maintenance of this inhibitory state requires dimeric proteins known as 14-3-3s. Phosphorylated CR2 (pS365 in BRAF) and C-terminal (pS729 in BRAF) regions serve as the binding sites for 14-3-3 proteins (43, 44). Nevertheless, KRAS is connected to BRAF in an autoinhibited state when bound to MEK1 and a 14-3-3 dimer in the form of pentameric KRAS/BRAF/MEK1/14-3-3 complex. KRAS is linked to the RAS-binding domain (RBD) of BRAF and exists in two different orientations. Experiments conducted to activate BRAF *in vitro* confirm that KRAS is insufficient for BRAF activation without membrane recruitment. The fundamental inhibitory interactions in the complex remain unchanged even with KRAS binding (**Figure 4B**) (42, 45).

## 4 Mechanistic inhibition of RAFs and MEKs

### 4.1 BRAF mutations

**Figure 5:**
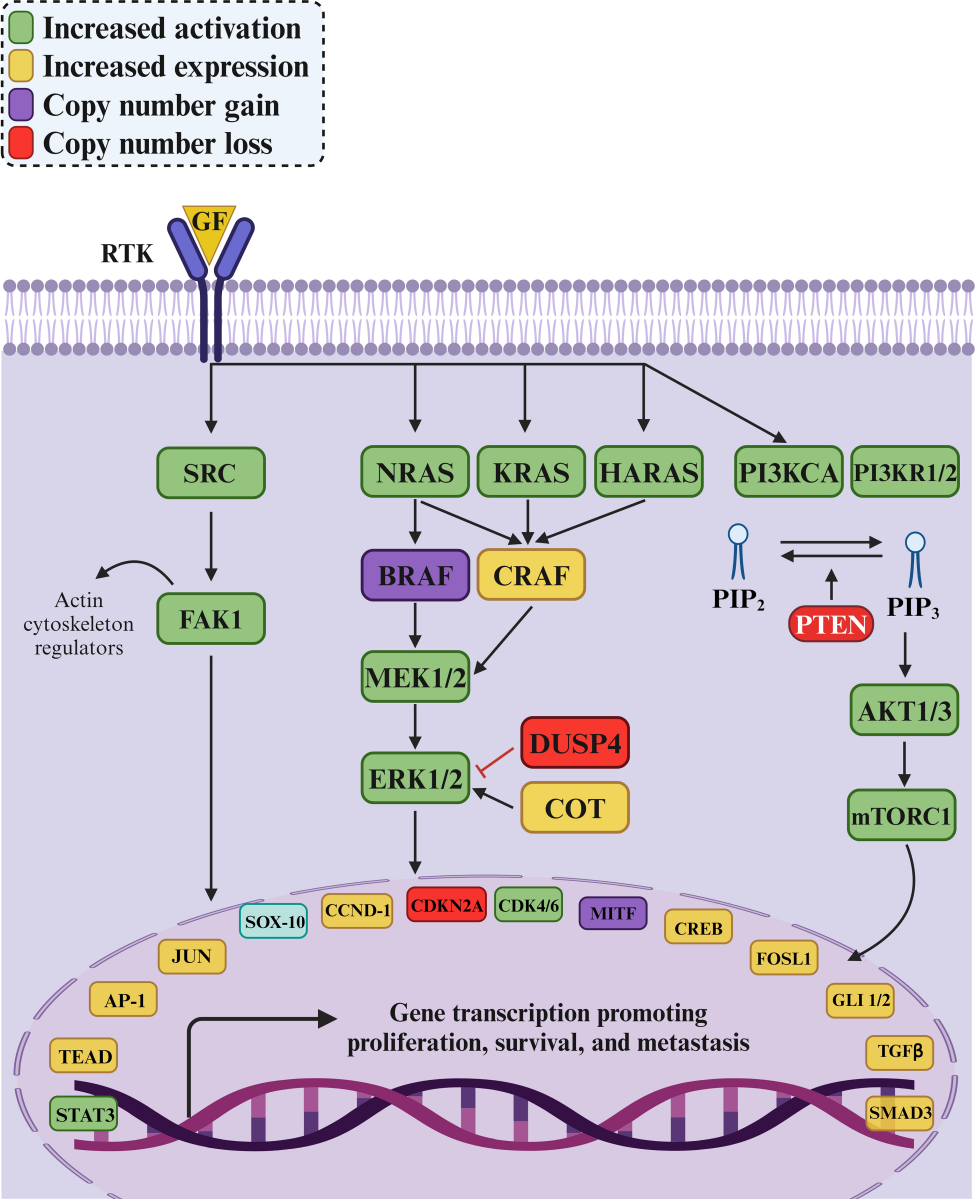
The MAPK/ERK signaling pathway and its role in cancer development. The pathway governs cell proliferation, survival, and metastasis through interactions among key proteins and enzymes like SRC, KRAS, BRAF, MEK1/2, and ERK1/2. Increased activation (green) and expression (yellow) of pathway components often lead to uncontrolled cell growth and cancer progression. Mutations, such as KRAS mutations in colorectal cancer and BRAF^V600E^ mutations in melanoma and colorectal cancer, hyperactivate the pathway, promoting tumorigenesis and metastasis. Loss of the tumor suppressor gene PTEN (red) activates the PI3K/AKT pathway, contributing to cancer progression by enhancing cell proliferation and survival. DUSP4, a negative regulator of ERK1/2, may experience copy number loss, diminishing its ability to control pathway activity. The intricate nature of the MAPK/ERK pathway and its crosstalk with other signaling pathways highlight the importance of targeted therapies to disrupt these interactions and effectively combat cancer.

The RAS/RAF/MAP signaling pathway is a critical regulator of cellular proliferation, survival, and metastasis, making it a frequent target for mutations in various cancers. These mutations can lead to aberrant pathway activation, driving uncontrolled cell growth and tumor development. The signaling cascade is initiated by growth factors binding to RTKs on the cell surface (28). This triggers RTK activation and subsequent autophosphorylation, creating docking sites for adaptor proteins like GRB2. GRB2, in turn, recruits SOS, a guanine nucleotide exchange factor that activates RAS proteins by facilitating the exchange of GDP for GTP. Activated RAS proteins (NRAS, KRAS, or HRAS) initiate a downstream signaling cascade, primarily by activating RAF kinases, including BRAF and CRAF. These kinases subsequently phosphorylate and activate MEK1/2, which then activates ERK1/2. Activated ERK1/2 translocates to the nucleus, phosphorylating and regulating transcription factors that control gene expression in cell cycle progression, survival, and metastasis (46, 47). These critical transcription factors include AP-1, TEAD, and STAT3, and their targets include genes like CCND1 and CDK4/6, which promote cell cycle progression (**Figure 5**). Therefore, mutations in various components of this pathway are frequently observed in cancer, leading to its dysregulation (48). Similarly, mutations in BRAF, such as the V600E mutation commonly found in melanoma, can also result in constitutive kinase activity and downstream pathway activation (49).

On the other hand, the PI3K/AKT pathway, a parallel signaling cascade often dysregulated in cancer, is also interconnected with the RAS/RAF/MAPK pathway. Mutations in PI3KCA and PI3KR1/2, which encode catalytic and regulatory subunits of PI3K, can lead to increased AKT activation, promoting cell growth and survival. Additionally, loss of PTEN, a tumor suppressor that negatively regulates PI3K/AKT signaling, is frequently observed in cancer and further contributes to pathway hyperactivation (50).

Furthermore, mutations in negative regulators of the RAS/RAF/MAPK pathway, such as DUSP4, which dephosphorylates and inactivates ERK1/2, can further enhance pathway signaling (51). When mutated, COT, a kinase capable of activating ERK1/2 independently of BRAF, can also contribute to pathway dysregulation. These alterations ultimately converge on the dysregulation of key transcription factors like SOX-10, JUN, MITF, and CREB, leading to the aberrant gene expression that drives oncogenesis (**Figure 5**) (52, 53).

BRAF mutations fall into three distinct classes, categorized by their specific effects. Most of these mutations are located in the kinase domain. Class I mutations exclusively consist of V600 missense mutations. In contrast, Classes II and III mutations exhibit a broader range of diversity, involving different positions and types of mutations, including missense mutations, insertions, deletions, insertion-deletions, and gene fusions. Interestingly, depending on the variant, certain positions can give rise to either class II or class III mutations. For instance, BRAF p.G469A is classified as a class II mutation, while BRAF p.G469E falls into the class III category (54, 55). Presently, there have been 373 reported BRAF mutations, with 76 of them associated with pathogenic effects, leading to either an increase or decrease in protein function. Out of these mutations, 52 mutations are located in the kinase domain. Classes II and III predominantly revolve around the active site, explicitly focusing on the region responsible for coordinating the phosphate tail with Mg^2+^. However, it is important to highlight two exceptions to these trends. Positions E549 and E586 likely participate in an unidentified mechanism involving another partner. Consequently, they were excluded from the dataset, resulting in 50 well-characterized mutations in the kinase domain: 26 were classified as class II and 24 as class III (56).

Patients with advanced melanoma can be categorized into two groups based on their BRAF mutations: V600 and non-V600. These distinct mutation classes can provide insights into how individuals will respond to targeted therapies, which has significant implications for future drug development (57). In a study involving 779 tumor cases, an automated immunohistochemistry (IHC) staining method using a mouse monoclonal anti-BRAF^V600E^ (VE1) primary antibody detected the presence of the BRAF^V600E^ mutation in 150 cases. These cases included 38 out of 611 colorectal carcinomas (approximately 6%), 102 out of 127 papillary thyroid carcinomas (about 80%), and 10 out of 41 malignant melanomas (around 24%) (58). In 2011, the FDA and EMA approved vemurafenib for treating metastatic melanoma with BRAF^V600^ mutations. While some research suggests potential benefits in continuing vemurafenib treatment after local therapy in certain patients experiencing progressive disease (PD), those who extended their vemurafenib treatment for more than 30 days following local therapy for PD lesions had an indeterminable median overall survival (OS). Patients who could not continue treatment with vemurafenib had a median OS of 1.4 months from the point of disease progression (59).

On the other hand, a combination of vemurafenib and cobimetinib (GDC-0973), a potent and highly selective inhibitor of MEK1/2, showed promise and completed a Phase III clinical trial (CoBRIM; ClinicalTrials.gov identifier: NCT01689519). This combination extended the median progression-free survival (PFS) for patients receiving cobimetinib and vemurafenib to 12.6 months (with a 95% confidence interval of 9.5-14.8), while those taking a placebo alongside vemurafenib had a median PFS of 7.2 months (with a 95% confidence interval of 5.6-7.5) (60). Additionally, dabrafenib (GSK2118436), designed for mutated BRAFs, and trametinib, a specific MEK 1/2 inhibitor, gained FDA approval in 2013 as individual treatments for metastatic melanoma with BRAF mutations. Their combined use has also received accelerated FDA approval. Both drugs target the MAPK pathway, with dabrafenib inhibiting mutant BRAF and trametinib selectively inhibiting MEK1 and MEK2 proteins activated by RAF kinases (61). Clinical studies have shown dabrafenib’s activity against a broader range of BRAF^V600E/K/D/R^ mutations (ClinicalTrials.gov identifier: NCT01928940, ClinicalTrials.gov identifier: NCT01682213).

The phase III COLUMBUS trial (ClinicalTrials.gov identifier: NCT01909453) provided a seven-year update on the long-term efficacy and safety of the combination therapy of encorafenib and binimetinib in treating BRAF^V600E/K^-mutant melanoma (62). The study compared the outcomes of 577 patients with locally advanced, unresectable, or metastatic melanoma harboring the BRAF^V600E/K^ mutation who were randomized to receive either encorafenib (450 mg once daily) combined with binimetinib (45 mg twice daily), encorafenib alone (300 mg once daily), or vemurafenib alone (960 mg twice daily). Results demonstrated that encorafenib and binimetinib significantly improved PFS and OS compared to the monotherapy groups. The combination therapy group also exhibited a higher objective response rate (ORR), indicating a greater proportion of patients achieving a partial or complete response to the treatment. The combination of encorafenib and binimetinib was generally well-tolerated, with adverse events consistent with previous reports and manageable side effects such as nausea, vomiting, diarrhea, arthralgia, and fatigue (≥30%) (62). The combination therapy was associated with a lower incidence of certain adverse events than the monotherapy groups, suggesting an improved safety profile. The study concludes that the encorafenib and binimetinib combination provides a durable and effective treatment option for patients with BRAF^V600E/K^-mutant melanoma, offering significant improvements in PFS and OS over monotherapy with vemurafenib or encorafenib. These findings reinforce the value of combination therapies in managing advanced melanoma and highlight the ongoing evolution of targeted therapies in oncology, especially for genetically defined subsets of cancer patients.

**Table 1** concisely overviews pivotal clinical trials evaluating BRAFi in melanoma patients, outlining key outcomes such as ORR, PFS, and OS.

**Table 1:** Key clinical trials assessing the efficacy of BRAFi in melanoma patients.

Clinical trial	ORR	Median PFS (Months)	Median OS (Months)	Treatment arms (number of patients)	Reference
BRIM-3	57% 9%	6.9 1.6	13.6 9.7	Vemurafenib^*^ Dacarbazine^⁑^	(63)
BREAK-3	50% 6%	6.9 2.7	20 15.6	Dabrafenib^*^ Dacarbazine	(64, 65)
METRIC	19% 5%	4.9 1.6	15.6 11.3	Trametinib^*^ Chemotherapy	(66, 67)
COMBI-v	68% 53%	12.1 7.3	26 17.8	Dabrafenib + Trametinib^†^ Vemurafenib	(68)
COMBI-d	70% 54%	10.2 8.8	25.8 18.7	Dabrafenib + Trametinib Dabrafenib	(69)
COMBI-i	68.5 64.2	16.2 12	Not Specified	Spartalizumab^‡^ + Dabrafenib + Trametinib Dabrafenib + Trametinib	(70)
coBRIM	70% 50%	12.6 7.2	22.5 17.4	Vemurafenib + Cobimetinib^†^ Vemurafenib	(71)
COLUMBUS	63% 51% 40%	14.9 9.6 7.3	33.6 23.5 16.9	Encorafenib^*^ + Binimetinib^†^ Encorafenib Vemurafenib	(72–74)

^*^BRAFi; ^⁑^Chemotherapy; ^†^MEKi; ^‡^Anti-programmed cell death protein-1 (PD-1).

This table summarizes key clinical trials investigating the roles of BRAFi in patients with melanoma, including the ORR, PFS, and OS outcomes.

As mentioned, BRAFi and MEKi have received approval for treating advanced melanoma with BRAF^V600^ mutations, achieving response rates as high as 70%. Furthermore, targeted therapy has demonstrated effectiveness in cases with various non-V600 BRAF mutations. Therefore, employing sensitive, precise, and comprehensive methods for detecting BRAF alterations is crucial to accurately match patients with the relevant, targeted treatments. Moreover, multiple BRAF alterations were detected in melanoma patients (75). The increased use of susceptible detection methods like next-generation sequencing has led to the discovery of various BRAF mutations beyond the V600E/K type in individuals with melanoma. In patients diagnosed with stage III or IV melanoma, non-V600 BRAF mutations such as V600R, V600_K601delinsE, K601E, p.T599_V600insT, L597V, G466R, S467L, and A598T were observed. BRAF^G466R^ and BRAF^A598T^ mutations were not previously documented in melanoma cases. Four of these patients received a combination of BRAFi/MEKi, two received BRAFi monotherapy, and six underwent treatment with immune checkpoint inhibitors (ICI) for advanced melanoma.

Additionally, four patients received adjuvant nivolumab, programmed cell death protein 1 (PD-1) inhibitor antibody (76). Selecting the first-line treatment for those with advanced-stage BRAF-mutant melanoma has posed challenges. Although BRAF-targeted therapy frequently generates higher response rates, ICIs typically provide more enduring responses (77).

Furthermore, in a multicenter study involving metastatic melanoma patients with well-defined BRAF mutations, 856 individuals were selected to analyze BRAF mutation patterns, their response to MAPK pathway inhibitors, and survival outcomes. Among these 856 patients, 51 (approximately 6%) had non-V600E/K BRAF mutations affecting codons V600 (24 out of 51, 47%, with V600G at 27.4% and V600R at 15.6%), K601 (6 out of 51, 11.7%), and L597 (4 out of 51, 7.8%). The study revealed an encouraging response to MAPK pathway inhibitors, such as BRAFi alone or in combination with MEKi, was observed in 56% (353 out of 631) of patients with V600E/K mutations with a median PFS of 7.7 months. Notably, the ORR was higher among patients treated with BRAFi and MEKi than those receiving BRAFi (78). However, limited efficacy data is available for patients with less common BRAF mutations. Individuals with uncommon BRAF mutations can exhibit a response to targeted treatment, although the effectiveness appears to be less pronounced when compared to V600E mutated melanoma. Combining BRAFi and MEKi offers the most promising treatment for V600 and non-V600 mutations (79).

### 4.2 Classification of BRAFi/MEKi

**Figure 6:**
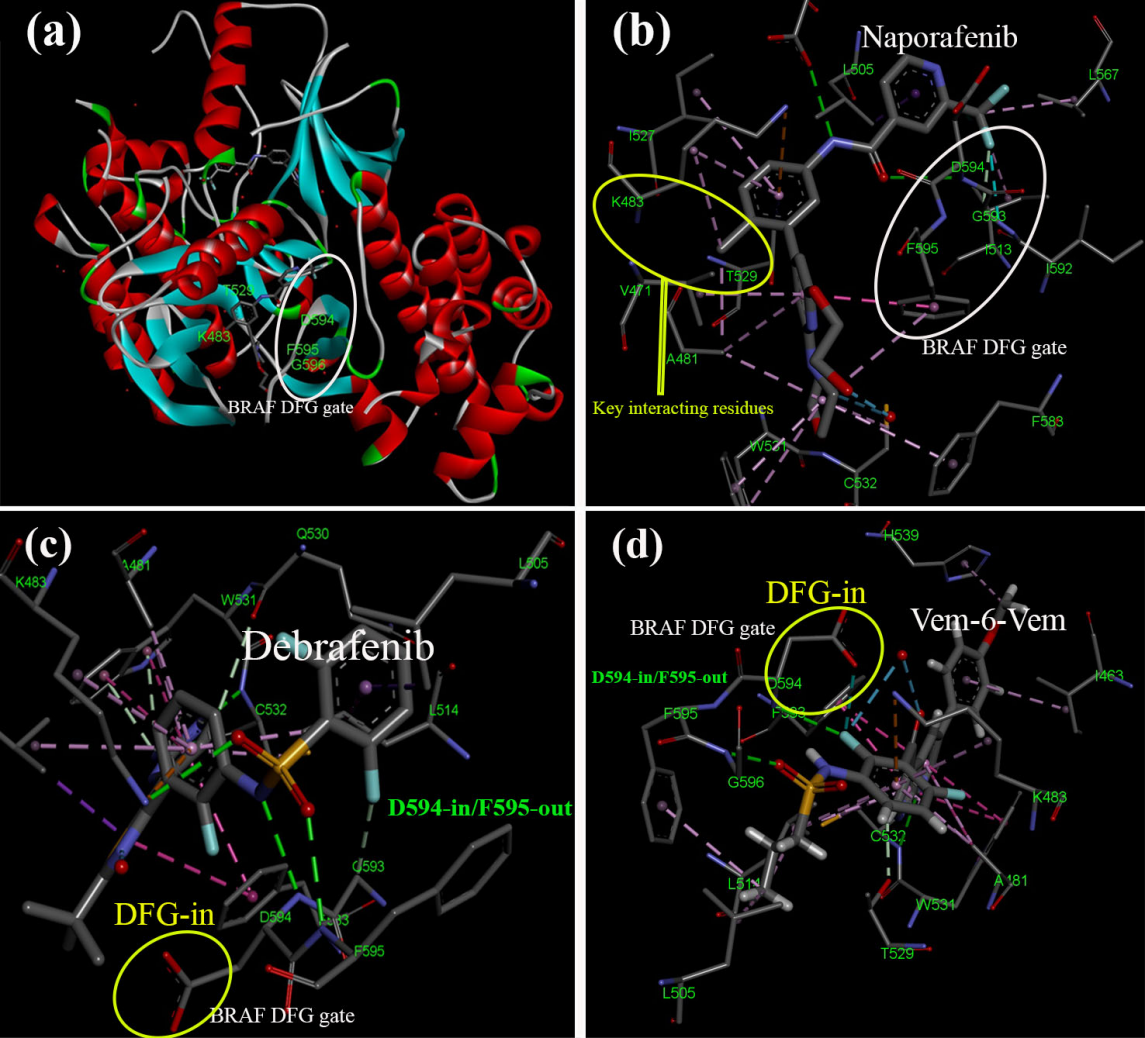
Structural insights into BRAF and interactions with BRAFi. The activation segment typically starts with a sequence of amino acid residues referred to as DFG, which signifies Aspartate (D), Phenylalanine (F), and Glycine (G). In the inactive state **(A, B)**, the phenylalanine side chain occupies the ATP-binding pocket. In contrast, the aspartate side chain points away from the active site, resulting in the DFG-out conformation. The type II BRAFi naporafenib binds to BRAF in its inactive form [PDB ID: 8F7P (80)]. In contrast, the active state **(C, D)** involves the phenylalanine side chain rotating out of the ATP-binding pocket, with the aspartate side chain turning inward to enter the ATP-binding pocket and coordinate with Mg^2+^, creating the DFG-in conformation. In this configuration, BRAF is bound to **(C)** dabrafenib [PDB ID: 4XV2 (81)] and **(D)** two chemically linked vemurafenib molecules [PDB ID: 5JRQ (82)].

Approved combinations of BRAFi and MEKi include dabrafenib/trametinib, vemurafenib/cobimetinib, and encorafenib/binimetinib (**Figure 6**) (83). The selectivity of these BRAF inhibitors for BRAF^V600E^ mutants is based on their binding mechanism (84). The binding of these inhibitors necessitates the outward movement of the C-helix, a conformation easily accessible in the monomeric state (85, 86). This binding mechanism is called “Type 1.5” to distinguish it from type I inhibitors, which also occupy the ATP site but do not require the C-helix-out conformation (87). When the RAF kinase domain forms dimers, it stabilizes the inward, active position of the C-helix, making it difficult for type 1.5 inhibitors to bind effectively. As a result, these agents are ineffective against RAF dimers and are termed RAF-monomer inhibitors (88, 89).

On the other hand, “Type II” RAF inhibitors have been formulated as potent suppressors of RAF dimers (80). A range of compounds that effectively inhibit RAF dimers has been developed. Most of these drugs follow a “Type II” binding pattern, which is characterized by a “DGF-out” configuration of the kinase (87). The DFG motif, a conserved segment consisting of three residues (Asp-Phe-Gly), is located at the beginning of the kinase activation loop. Type II inhibitors interact with or induce a conformational change involving a rotational flip of the DFG segment. This repositioning redirects the phenylalanine residue toward the ATP site (90). Inhibitors following a type II binding mode extend from the ATP site and insert a hydrophobic group into the region vacated by the DFG phenylalanine. Tovorafenib (DAY101), TAK-580, and naporafenib (LXH254) are examples of type II inhibitors currently undergoing clinical development (91, 92).

Prominent examples of type II inhibitors in clinical development include tovorafenib (DAY101 and TAK-580) and naporafenib (LXH254) (91, 92). These compounds have demonstrated their potential to effectively inhibit RAF dimers and represent a promising avenue in cancer treatment. The pyrimidine ring of tovorafenib establishes two critical hydrogen bonds with the kinase hinge: one with the backbone amide of C532 and another with its carbonyl group. Moreover, tovorafenib’s trifluoromethyl-substituted pyridine ring fits neatly within a hydrophobic pocket left vacant by DFG phenylalanine (F595), forming critical interactions. Similarly, naporafenib interacts with specific amino acid positions, with its central phenyl ring occupying the space between T529 and K483 and its trifluoromethyl pyridyl moiety nestled in the hydrophobic pocket (80).

However, the complexities of RAF kinase inhibitors extend beyond their binding mechanisms. The formation of homo- and hetero-dimers involving different RAF isoforms has led to paradoxical activation effects. Inactive RAF kinase domains do not form dimers, but several BRAFi disrupt this autoinhibited complex, unexpectedly facilitating the activation of the partner kinase in the dimer. These inhibitors stabilize the active state in the partner kinase, which is accessible from the inhibitor when present at sub-saturating concentrations (93).

An intriguing approach to addressing these challenges is using the allosteric characteristics of RAFi and MEKi. Allosteric BRAFi has shown potential in disrupting BRAF dimers and countering overactive MAPK signaling resulting from oncogenic BRAF or RAS mutations. Computational methods have been employed to design peptide-based inhibitors, such as braftide, targeting the dimer interface of BRAF. These inhibitors have exhibited strong effectiveness in inhibiting the kinase activity of both BRAF homodimers and heterodimers (94). Additionally, ponatinib, an FDA-approved drug, can inhibit BRAF monomers and dimers. Ponatinib binds to the BRAF dimer and causes a specific change in the αC-helix, an essential part of the protein’s structure. These structural insights have led to the development of ponatinib hybrid inhibitor 1 (PHI1), a novel inhibitor selectively targeting BRAF dimers inside cells (95). MEKi also plays a crucial role in this intricate network and binds to allosteric sites, interacting with specific amino acid residues in MEK through which critical structural elements, such as the activation loop, are stabilized. This conformational change locks MEK into a unique state necessary for its proper inhibition (96).

However, a new class of RAF inhibitors, referred to as “paradox breaker” inhibitors, presents a promising advancement in the field. Notable examples include PLX7904, PLX7922, and PLX5568. These compounds have shown the capability to disrupt RAF dimerization. In addition, PLX7904 and its optimized version, PLX8394, share a structural resemblance to vemurafenib. Analyzing the crystal structure of PLX7904 bound to BRAF^V600E^ revealed a binding mode akin to vemurafenib. Importantly, the N-ethyl methyl group of PLX7904 occupies the same internal pocket as vemurafenib’s propyl group but forms closer interactions with Leu505, a key kinase regulatory residue in the αC helix (81).

Additionally, PLX4032 (RG7204), an effective BRAFi, exhibited substantial inhibitory effects on the RAF/MEK/ERK pathway in cells with BRAF mutations. PLX4032 binds to one of the protomers in its crystal structure, inducing the DFG-in conformation. This binding forms a unique hydrogen bond between D594’s backbone NH and PLX4032’s sulfonamide nitrogen. Furthermore, PLX4032 causes an outward shift in the regulatory αC helix, likely contributing to its distinct impact on RAF dimerization compared to other inhibitors like AZD-628 and GDC-0879. These findings highlight the potential of these inhibitors in targeting RAF kinases with novel mechanisms of action (97).

These discoveries highlight the diverse strategies and approaches in targeting RAF kinase inhibition, with potential implications for cancer therapy. From Type I and Type II inhibitors to allosteric inhibitors and “paradox breaker” compounds, these advancements are reshaping our understanding of kinase regulation and offering new avenues for precision medicine in cancer treatment.

## 5 FDA-approved BRAF inhibitors – clinical background

### 5.1 Vemurafenib

#### 5.1.1 Vemurafenib in BRAF-mutated melanoma treatment

Approximately half of melanoma cases involve BRAF^V600^ mutations, and early trials indicated promise for vemurafenib, an oral BRAFi. A phase II trial enrolled 132 patients, examining how vemurafenib influenced tumor response rates, response duration, and OS (ClinicalTrials.gov identifier: NCT00949702). Results revealed a confirmed response rate of 53%, with 6% achieving a complete response and 47% a partial response. Responses typically endured for around 6.7 months, and PFS averaged about 6.8 months. Only 14% of patients experienced primary progression, and some maintained their response even after six months of vemurafenib treatment. Median OS reached roughly 15.9 months. In this study, with a substantial follow-up period, the median OS reached about 16 months, and it was indicated that vemurafenib delivered clinical benefits to over half of the previously treated metastatic melanoma patients with BRAF^V600^ mutations (98).

#### 5.1.2 Vemurafenib versus dacarbazine

Vemurafenib demonstrated a strong response in over 50% of patients with metastatic melanoma with BRAF^V600E^ mutation. In a phase III randomized trial comparing vemurafenib to dacarbazine (an alkylating chemotherapy) in 675 previously untreated metastatic melanoma patients with the BRAF^V600E^ mutation, vemurafenib showed remarkable results (ClinicalTrials.gov identifier: NCT01006980). At the 6-month, vemurafenib achieved an 84% OS rate, while dacarbazine yielded 64%. Response rates were notably higher, with vemurafenib at 48% compared to dacarbazine’s 5% (99).

#### 5.1.3 Subgroup Analysis, BRAF^V600E^ and BRAF^V600K^ Mutations

The impact of vemurafenib and dacarbazine on patients with advanced melanoma carrying BRAF^V600^ mutations, explicitly focusing on the BRAF^V600E^ and BRAF^V600K^ mutation subgroups, was thoroughly investigated. In the context of patients with BRAF^V600E^ disease, constituting 91% of the total, vemurafenib exhibited a significant advantage in terms of both OS (13.3 months compared to 10.0 months for dacarbazine) and PFS (6.9 months compared to 1.6 months). For the smaller subgroup with BRAF^V600K^ disease, comprising 9% of the total, vemurafenib showcased a substantial improvement in both OS (14.5 months compared to 7.6 months with dacarbazine) and PFS (5.9 months compared to 1.7 months) (63).

#### 5.1.4 Long-Term Vemurafenib Safety

Vemurafenib showed effectiveness and safety in a diverse group of patients with specific genetic mutations in advanced melanoma (ClinicalTrials.gov identifier: NCT01307397). These results were consistent with earlier drug studies. After a two-year monitoring period, the safety of long-term vemurafenib treatment remained consistent among a substantial cohort of patients (N=3219) afflicted with metastatic melanoma carrying the BRAF^V600^ mutations. This patient group represents a more real-world clinical practice scenario, differing from the usual clinical trial populations. These findings indicate that extended vemurafenib therapy is both practical and well-tolerated, with no emergence of new safety concerns (100).

#### 5.1.5 Vemurafenib combined with cobimetinib MEKi

The combination of MEKi with BRAFi was also clinically tested for patients with melanoma. Combining a MEKi with a BRAFi has shown enhanced efficacy in inhibiting tumor growth, delaying acquired resistance development, and eliminating paradoxical activation of the MAPK pathway in preclinical models of BRAF-mutated melanoma. A randomized phase III clinical trial was investigated concurrently using the vemurafenib and the MEKi cobimetinib (ClinicalTrials.gov identifier: NCT01271803). Administering vemurafenib and cobimetinib at their maximum tolerated doses proved safe and well-tolerated. This combination therapy displayed promising anti-tumor activity, particularly in patients with advanced BRAF(V600)-mutated melanoma without a BRAFi (101).

Another Phase III study indicated that adding cobimetinib to vemurafenib significantly improved PFS in patients with metastatic melanoma harboring the BRAF^V600E^ mutations. However, this improvement was accompanied by a slight increase in treatment-related side effects (ClinicalTrials.gov identifier: NCT01689519). In this study involving 495 previously untreated melanoma patients with the BRAF^V600^ mutations, researchers assessed the combination of vemurafenib and cobimetinib versus vemurafenib alone. The combination therapy resulted in significantly prolonged PFS (9.9 months vs. 6.2 months) and higher rates of complete or partial responses (68% vs. 45%). Furthermore, the combination therapy demonstrated better nine-month survival (81% vs. 73%) (102).

#### 5.1.6 Combination of Vemurafenib with Immune Cell Therapy

In parallel, innovative approaches like combining vemurafenib with tumor-infiltrating lymphocytes (TILs) demonstrated exciting clinical responses, offering new avenues for melanoma research. A pilot clinical trial demonstrated the safety and feasibility of administering vemurafenib in combination with TILs to treat metastatic melanoma. This treatment approach was well-tolerated and exhibited a safety profile like TIL or vemurafenib alone. Remarkably, 64% of patients achieved an objective clinical response, with 18% of complete responses for up to three years. *In vitro* studies revealed that vemurafenib could inhibit the proliferation and viability of TILs and peripheral blood T cells. However, the T cell receptor repertoire and the ability of T cells to recognize autologous tumors remained unchanged between pre- and post-vemurafenib treatment (103).

These findings collectively support vemurafenib as a cornerstone in treating BRAF-mutated melanoma, offering hope and improved outcomes for patients facing this challenging disease. Vemurafenib was among the pioneering BRAF inhibitors approved for treating BRAF-mutated melanoma. It has demonstrated significant efficacy in clinical trials, with notable benefits such as high tumor response rates and improved OS. Vemurafenib has also been explored in combination therapies with MEKi, such as cobimetinib, which has enhanced anti-tumor activity. However, it is worth noting that some patients on vemurafenib have experienced side effects, including the development of secondary cutaneous cancers.

### 5.2 Dabrafenib

#### 5.2.1 Dabrafenib in BRAF-mutated melanoma treatment

Dabrafenib (GSK2118436) has demonstrated significant efficacy as an anticancer drug, particularly benefiting patients with melanoma characterized by BRAF gene mutations. A preliminary clinical study evaluated 76 patients with BRAF^V600E^ melanoma and 16 patients with BRAF^V600K^ melanoma mutations. Among those with BRAF^V600E^, 45 patients (59%) exhibited a confirmed response, including five patients (7%) with complete responses. In contrast, two patients (13%) with BRAF^V600K^ mutation had confirmed partial responses. Importantly, baseline cfDNA levels proved predictive of response rate and PFS in patients with BRAF^V600E^ melanoma mutations (104).

#### 5.2.2 Dabrafenib combined with Trametinib MEKi

Trametinib, a MEKi, has significantly improved chemotherapy in patients with metastatic melanoma with BRAF^V600E/K^ mutations (67). Consequently, the combination of dabrafenib and trametinib, when compared to dabrafenib monotherapy, demonstrated significant enhancements in PFS and overall response rates for previously untreated metastatic melanoma patients with BRAF^V600E/K^ mutations (ClinicalTrials.gov identifier: NCT01584648). The study revealed a median PFS of 9.3 months for the combination group, representing a 25% reduction in the risk of progression or death compared to dabrafenib alone. The overall response rate was also higher in the combination group (67%) compared to the dabrafenib-only group (51%) (105).

In a separate phase III study involving patients with advanced stage IIIC/IV metastatic melanoma (ClinicalTrials.gov identifier: NCT01584648), the advantages of combining dabrafenib with trametinib were once again demonstrated, leading to prolonged OS compared to using dabrafenib alone (69). Notably, circulating tumor DNA (ctDNA) emerged as a potential biomarker for predicting patient survival in those receiving dabrafenib and trametinib combination therapy. Detectable ctDNA levels have correlated with poorer outcomes, particularly in cases with elevated lactate dehydrogenase levels (106).

Despite advancements in adjuvant melanoma therapy, early recurrence remains a significant challenge in clinical practice. A retrospective multicenter study examined stage III-IV melanoma patients treated with adjuvant nivolumab, pembrolizumab, or dabrafenib+trametinib, estimating the 12-month recurrence-free survival (RFS). The study findings indicate that total lymph node dissection does not decrease the risk of early melanoma recurrence and should be considered only in specific cases. While PD-1 blockade emerged prominently in adjuvant melanoma therapy, all available adjuvant treatments for high-risk melanoma patients offer value, broadening treatment choices. Comparisons among these studies suggest that although adjuvant BRAF+MEK inhibition demonstrated improved 12-month RFS, differences in RFS diminish over time (107).

The approach to treating BRAF-mutated melanoma still lacks effectiveness despite the advancement in prognosis for advanced melanoma due to immune checkpoint inhibition. Targeted therapy swiftly manages the disease in many patients, but the emergence of secondary resistance shortens response duration. On the other hand, immunotherapy might trigger slower yet longer-lasting responses in specific patient groups. Hence, finding a combined approach using these therapies holds promise. Presently, varying data exist, yet most studies suggest that administering BRAFi/MEKi before ICIs potentially diminishes the effectiveness of immunotherapy (108).

On the other hand, discontinuation of dabrafenib and trametinib due to treatment-related adverse events (TRAEs) of any severity stood at 9%. Other reasons for stopping treatment included patient-driven decisions (6%), decisions by physicians (6%), adverse events unrelated to treatment (3%), disease progression (5%), and various other causes (5%). The median duration until treatment discontinuation was nine months. Severe (Grade 3-4) TRAEs occurred in 21.5% of patients, with the most prevalent being fever (3%), fatigue (3%), and diarrhea (3%). Unplanned hospitalizations and clinical examinations were observed in 6% and 22% of patients. Over a median follow-up of 20 months (with a 95% CI of 18-22), disease progression led to the passing of 9% of patients, while the 12-month rates for relapse-free survival and OS stood at 95.3% and 100%, respectively (109).

Although treating BRAF-mutant melanoma with BRAF and MEK inhibition has shown effectiveness despite notable treatment-related side effects, the scrutiny of drug-drug interactions impacting the toxicity linked to anti-BRAF/anti-MEK therapy has become imperative. These interactions are especially concerning due to their potential impact on treatment-related cardiovascular toxicity. Understanding and addressing drug-drug interactions, as a critical safety concern and a pivotal theme in precision medical oncology, are essential to promote optimal adherence to cancer treatment and reduce associated toxicities, notably the notably heightened risk of cardiovascular complications (110).

#### 5.2.3 Dabrafenib Approvals

Finally, the series of approvals, from monotherapy to combination treatments, reflects the growing recognition of dabrafenib’s significance in melanoma treatment. These milestones signify a continued commitment to advancing targeted therapies, providing new avenues of hope for patients facing this challenging disease. In the United States, dabrafenib received the first global approval in 2013 as a monotherapy for patients with the BRAF^V600E^ mutation and unresectable or metastatic melanoma (111). In 2018, the FDA formally authorized dabrafenib (TAFINLAR, Novartis) and trametinib (MEKI NIST, Novartis) as supplementary treatments for individuals with melanoma with BRAF^V600E^ or BRAF^V600K^ genetic mutations. Furthermore, on June 22, 2022, the FDA approved the combination of dabrafenib and trametinib to treat patients aged six and older with advanced solid tumors carrying the BRAF^V600E^ mutation (112). These approvals signify the continued advancement of targeted therapies in melanoma treatment.

### 5.3 Encorafenib

#### 5.3.1 Encorafenib combined with Binimetinib MEKi

Encorafenib is another medication explicitly targeting tumors with the BRAF^V600E^ mutation. A recent phase Ib/II study investigated the combination of encorafenib (BRAFi) and binimetinib (MEKi), two inhibitors of the BRAF pathway, in patients with solid tumors carrying this mutation (ClinicalTrials.gov identifier: NCT01543698). Notable responses were observed in phase II, with 18% in metastatic colorectal cancer, 42% in anti-BRAF-pretreated melanoma, and 67% in treatment-naïve melanoma. This combination therapy displayed manageable side effects and promising activity in patients with BRAF^V600E^-mutant tumors. Its safety profile was consistent with other approved BRAFi plus MEKi regimens, with some differences, including lower rates of specific adverse events like fever, joint pain, and photosensitivity (113).

Another clinical study compared the combination of encorafenib and binimetinib with vemurafenib (ClinicalTrials.gov identifier: NCT01909453). In this study, 577 out of 1345 screened patients were randomly assigned to receive either encorafenib plus binimetinib (192 patients), encorafenib alone (194 patients) or vemurafenib (191 patients). The median follow-up period was 16.6 months. Encorafenib plus binimetinib demonstrated superior PFS with a median of 14.9 months compared to 7.3 months for vemurafenib. There were no treatment-related deaths, except one possibly related to treatment in the combination group. Therefore, encorafenib plus binimetinib showed improved efficacy and a better tolerability profile than encorafenib or vemurafenib, offering a potential treatment option for patients with BRAF-mutant melanoma (72). After a five-year follow-up, the median duration of response in the encorafenib plus binimetinib group was 18.6 months, and disease control was achieved in 92.2% of patients (114).

Both trials explored the combination of encorafenib and binimetinib, showing promising results in patients with BRAF^V600E^ tumors. The second trial compared this combination to vemurafenib, demonstrating its superiority in terms of PFS. These findings offer potential treatment options for patients with BRAF^V600E^ melanoma, which reached FDA approval in 2018 (115).

## 6 BRAF/MEK inhibitors in melanoma brain metastases

Brain metastasis is a common and severe complication in melanoma patients with BRAF and NRAS mutations, often resulting in a poor prognosis. While BRAF inhibitors have been approved for the treatment of melanoma, their limited ability to cross the blood-brain barrier restricts their effectiveness against brain metastases. The presence of brain metastases signifies disease progression in a substantial portion of melanoma patients, presenting a significant challenge to treatment efficacy. Understanding the mechanisms underlying the development and maintenance of melanoma brain metastases is critical for innovating new treatment strategies. Consequently, there is a vital need for enhanced treatments targeting melanoma brain metastasis.

Vemurafenib has shown activity in patients with BRAF^V600^ mutation-positive melanoma and brain metastases. Despite this, many patients in a phase II clinical trial yield to disease progression. The study indicated that vemurafenib could achieve clinically meaningful responses in melanoma brain metastases while maintaining a tolerable safety profile and without substantial central nervous system (CNS) toxicity (116). Challenges in accurately measuring brain metastases likely caused discrepancies between investigator assessments and independent review committee determinations. The data suggest that brain metastases in BRAF-mutant melanoma are less responsive to BRAF inhibition. This reduced responsiveness may be due to different tumor characteristics of brain metastases, varying characteristics between BM and extracranial metastases in patients with brain metastases, or differences in drug concentrations between intracranial and extracranial metastases.

In addition, the role of lactate dehydrogenase (LDH) level as a biomarker for patients receiving the combination of dabrafenib with trametinib was also reported (117). Brain metastases and LDH levels above the normal range are linked to poor prognosis in melanoma patients. While the combination treatment of the BRAFi dabrafenib and the MEKi trametinib has displayed prolonged clinical advantages in melanoma patients, there is limited data on their effectiveness in those with brain metastases. In this analysis, which focused on 325 assessable patients receiving first-line therapy, 76 patients (23.4%) had brain metastases at the study’s outset. The median PFS was initially shorter for patients with brain metastases than the overall patient population (8.7 months vs. 9.3 months, respectively). Patients diagnosed with brain metastases and elevated LDH levels experienced notably shorter mPFS compared to those with LDH levels in the normal range (118). The findings support the efficacy of dabrafenib plus trametinib in a real-world setting among patients with advanced BRAFV600-mutated melanoma and baseline brain metastases, indicating its potential utility in this group with typically poor outcomes (118).

In parallel, belvarafenib, a pan-RAF inhibitor, has been shown to encourage anticancer activity in preclinical melanoma models and patients with BRAF and NRAS mutations. Nonetheless, additional studies are necessary to verify its ability to penetrate the brain and its effectiveness against brain metastases. Belvarafenib exhibited robust melanoma growth suppression in mice with BRAF^V600E^ mutations and significantly inhibited tumor progression in mice with NRAS mutations (119). Additionally, it showed enhanced anticancer effects when combined with cobimetinib or atezolizumab. Pharmacokinetic studies revealed that orally administered belvarafenib achieved significant concentrations in the brains of mice and rats, with brain levels comparable to or exceeding those in the blood. Belvarafenib’s effective brain penetration sets it apart from other BRAF inhibitors, which generally show poor ability to penetrate the brain. Significantly, belvarafenib substantially decreased tumor size and enhanced survival in mice with intracranially implanted A375SM melanoma cells. These results highlight the potential of belvarafenib as a promising therapy for patients with BRAF/NRAS mutant melanoma brain metastasis due to its capability to cross the blood-brain barrier and its anticancer solid effects (119).

Furthermore, the POLARIS phase II study (ClinicalTrials.gov identifier: NCT03911869) evaluated the combination of encorafenib and binimetinib in patients with BRAF^V600^-mutant melanoma and asymptomatic brain metastases who had not previously received BRAF/MEK inhibitors. The results indicated a 60% brain metastasis response rate among evaluable patients, with a 67% brain metastasis response rate in the phase II cohort, demonstrating a promising outlook for this combination therapy. The safety profile was consistent with previous reports of standard-dose encorafenib combined with binimetinib (120).

Another study analyzed transcriptome and methylome profiles of melanoma brain metastases with varying tumor-associated microglia and macrophages (TAMs) levels. Prognostic markers such as Amyloid beta A4 precursor protein-binding family B member 1-interacting protein (APBB1IP) and the interferon-responsive gene ITGB7 were identified, suggesting a favorable disease course and response to ICI therapy (121). Cases with elevated ITGB7/APBB1IP levels displayed a significant association between TAM presence and immune score. Signature-based deconvolution analysis revealed enrichment of interferon-response and immune signatures in melanoma brain metastasis samples, highlighting pathways related to inflammation, stress, and c-MET receptor signaling. Activation of the c-MET in brain-colonizing melanoma cells was found to promote tumor growth, potentially counteracting the effects of ICI therapy. Targeting the c-MET with inhibitors such as PHA-665752 and ARQ197 (tivantinib) demonstrated significant responses in brain metastasis-derived cell lines *in vivo*, suggesting the potential of MET-targeted therapy in managing melanoma brain metastases and improving patient outcomes (121).

Collectively, these findings illustrate the evolving landscape of melanoma treatment, particularly for patients with brain metastases. The challenges posed by brain metastases necessitate a multifaceted approach, integrating advanced targeted therapies like BRAF/MEK inhibitors and novel agents such as belvarafenib and leveraging the profound impact of immune checkpoint inhibitors. Continued research and clinical trials are paramount to optimizing these therapeutic strategies, improving brain penetration, and enhancing patient outcomes.

## 7 BRAFi/MEKi combined with immunotherapy

Researchers have a growing emphasis on combining BRAFi, which targets specific genetic mutations in melanoma, with immunotherapy in the treatment of melanoma (122). This approach seeks to optimize treatment efficacy by simultaneously targeting cancer cells directly and enhancing the body’s immune response against the disease. For example, the SECOMBIT trial (ClinicalTrials.gov identifier: NCT02631447) enrolled participants from various countries with untreated advanced melanoma characterized by the BRAF^V600^ genetic mutation. These individuals were categorized into three arms of the trial: Arm A, where patients initially received encorafenib plus binimetinib, followed by ipilimumab, a CTLA-4 inhibitor, plus nivolumab, a PD-1 inhibitor; Arm B, where patients started receiving ipilimumab plus nivolumab and subsequently received encorafenib plus binimetinib; and Arm C, where patients were initially administered with encorafenib plus binimetinib, followed by ipilimumab plus nivolumab, and then returned to encorafenib plus binimetinib. The study included a total of 209 patients. After an average follow-up period of approximately 32 months, none of the groups reached a median OS, signifying prolonged survival for all participants. All three groups exhibited favorable 2-year and 3-year survival rates, ranging from 54% to 73% (123). Furthermore, no newly identified safety concerns arose. Intriguingly, this research underscores that the combination of sequential immunotherapy and targeted therapy confers substantial survival advantages to individuals with BRAF^V600-mutant^ melanoma (123).

Conversely, the ImmunoCobiVem study (ClinicalTrials.gov identifier: NCT02902029) aimed to investigate atezolizumab, a PD-L1 inhibitor, after initial doses of vemurafenib plus cobimetinib for advanced melanoma with the BRAF^V600^ mutation to achieve more prolonged survival. Switching to atezolizumab after three months led to rapid disease progression. However, it offered a potential survival benefit after two years compared to staying on the initial targeted therapy (124). On the other hand, in the extended follow-up of the IMspire150 trial (ClinicalTrials.gov identifier: NCT02908672), it was found that there was no significant increase in OS when using atezolizumab, vemurafenib, and cobimetinib compared to vemurafenib and cobimetinib alone for patients with advanced melanoma carrying the BRAF^V600^ mutation (125).

**Figure 7:**
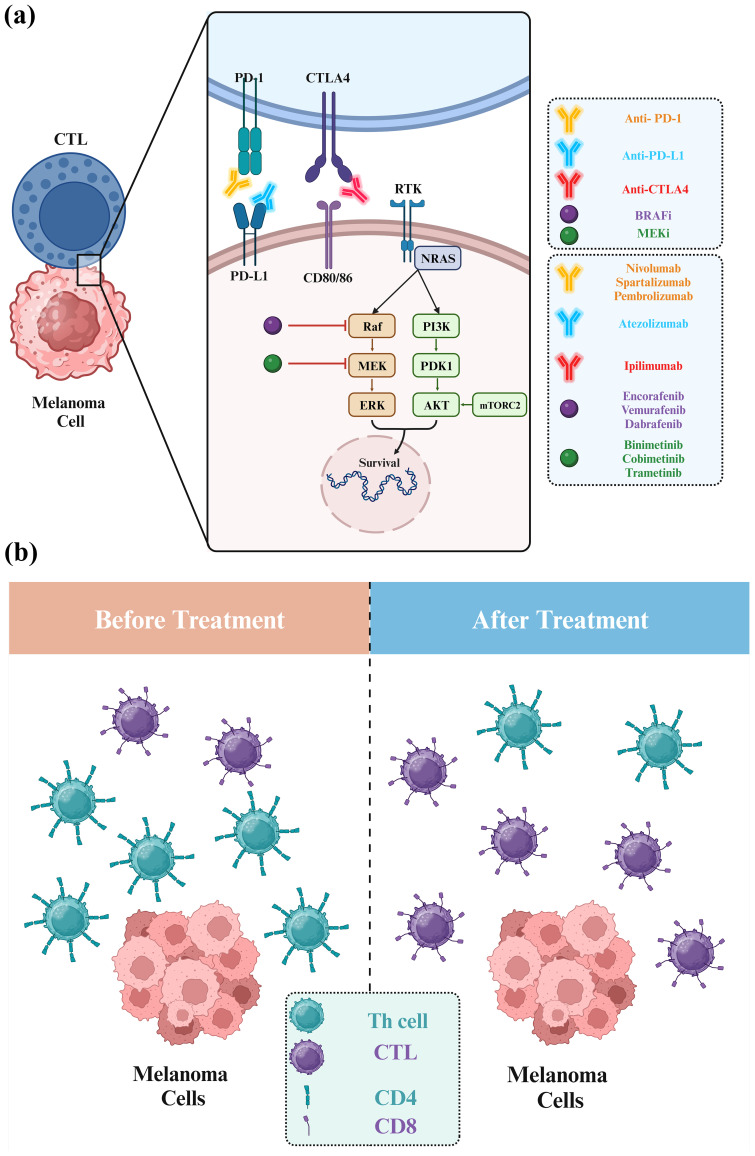
Combination therapy of melanoma using BRAF, MEK, and immune checkpoint inhibitors. **(A)** In melanoma, triple therapy integrates BRAFi, MEKi, and immune checkpoint inhibitors (ICI) to combat cancer cells via diverse pathways. BRAFi and MEKi disrupt cancer cell signaling, while ICI boosts the anticancer immune system. This strategy enhances treatment efficacy, overcomes resistance, and potentially elevates outcomes for advanced melanoma patients. **(B)** BRAF mutations in untreated melanoma shape distinct immune environments, marked by reduced CD8^+^ T cells and elevated CD4^+^ T cells. Metastatic BRAF-mutant melanomas display elevated CD4^+^ T and B cells but reduced CD8^+^ T cells.

Although administering anti-PD1 or trametinib for melanoma adjuvant therapy was effective, the results suggest a shift toward a less aggressive surgical approach in melanoma treatment (126). Nevertheless, the final analysis to determine whether long-term treatment with this combination of three drugs can result in a meaningful OS improvement compared to using only vemurafenib plus cobimetinib is pending (125). Similarly, preclinical research suggests combining an anti-PD-1 antibody with dabrafenib and trametinib is more effective against tumors than using dabrafenib and trametinib alone. This concept is supported by evidence demonstrating that combining ICIs with targeted therapy could enhance treatment outcomes for patients with BRAF^V600-mutant^ metastatic melanoma. In the COMBI-i phase III trial (ClinicalTrials.gov identifier: NCT02967692), spartalizumab, an anti-PD-1 antibody, was tested in combination with dabrafenib and trametinib in patients with unresectable or metastatic melanoma carrying the BRAF^V600^ mutations. Unfortunately, the study did not achieve its primary objective, and therefore, using spartalizumab-dabrafenib-trametinib as a first-line treatment for all patients is not recommended based on these findings. Further research may help identify specific patient groups who could benefit from combining ICIs and targeted therapy (**Figure 7A**) (70).

In a study from June 2016 to August 2018 involving 33 advanced melanoma patients, combining pembrolizumab with dabrafenib and trametinib displayed superior efficacy over pembrolizumab alone. Adverse effects (Grade 3-4 TRAE) varied among cohorts (12%, 12%, 50%, and 63%), and planned targeted therapy rates differed (88%, 63%, and 38% in cohorts 2, 3, and 4). Cohort 4 exhibited lower ORR at weeks 6 and 18. Median PFS was 10.6 months for pembrolizumab alone and unreached for combination therapy. Landmark PFS rates varied at 2 and 3 years. The combination therapy was better tolerated and manageable than continuous triple therapy (127).

Furthermore, in a cohort study of advanced cutaneous melanoma patients, exposure to immune checkpoint inhibitors or BRAFi/MEKi increased the risk of uveitis compared to the general population. These findings highlight a heightened uveitis risk with immune checkpoint therapy and BRAF/MEK targeted therapy, emphasizing the need for ocular monitoring during treatment (128). Patients with advanced melanoma treated with immune checkpoint inhibitors may experience ongoing disease control after treatment discontinuation without subsequent systemic anticancer therapy (129).

In metastatic melanoma patients with BRAF^V600E/K^ mutations, first-line immune checkpoint inhibitors showed superior survival outcomes compared to BRAFi/MEKi. A retrospective study of 40 patients receiving BRAFi/MEKi post-immunotherapy found a median OS of 20.3 months (130). Additionally, in advanced BRAF wild-type melanoma patients, a retrospective study comparing dual ICI with single ICI initially suggested better OS. Dual ICI exhibited more frequent and severe immune-related adverse events, necessitating increased systemic corticosteroid use compared to single ICI. While limited by the study’s retrospective nature and small sample size, a non-significant trend towards improved OS with dual ICI in BRAF V600 wild-type advanced melanoma was observed. Further research is needed to validate these findings (131).

Furthermore, among advanced melanoma patients treated with first-line ICI, NRAS mutations were commonly observed, primarily Q61R and Q61K (present in 49% of 637 patients) (**Figure 7A**). However, NRAS status did not significantly affect PFS or OS with either anti-PD1 monotherapy or anti-PD1 plus anti-CTLA4 therapy. ORRs were similar between NRAS-mutated and wild-type patients, with no correlation between NRAS mutations and PD-L1 expression (>5%). Factors such as high lactate dehydrogenase and brain metastases were associated with increased mortality risk (132). Another investigation using Dutch Melanoma Treatment Registry data from 2012 to 2021 explored the effect of genetic mutations on ICIs in advanced melanoma. Among 1764 patients receiving anti-PD-1 and 759 undergoing ipilimumab-nivolumab therapy, no significant distinctions were observed with anti-PD-1. However, ipilimumab-nivolumab illustrated extended median PFS in BRAF-mutant (9.9 months) compared to NRAS-mutant (4.8 months). Ipilimumab-nivolumab showed advantages for BRAF mutations, implying their relevance in selecting between single or combined checkpoint inhibition for advanced melanoma therapy (133).

The landscape of malignant melanoma treatments has undergone substantial changes in recent years. However, disparities between clinical trials and actual clinical practice are inevitable, owing to various patient-specific factors such as prior adjuvant therapy efficacy, diverse metastatic lesions, including brain metastases, and existing medical conditions (134). The debate over selecting ICIs and targeted therapies as the first-line approach has been ongoing. Notably, the outcomes from two significant clinical trials, the DREAMseq trial (135) and the SECOMBIT trial (123), have recently been published. Both trials showcased improved OS in melanoma patients treated with a first-line combination of nivolumab and ipilimumab therapy. The Kaplan-Meier OS curves observed in these trials reflect the distinct characteristics of targeted therapies, displaying a high response rate but short response duration, and ICIs, exhibiting a relatively low response rate but prolonged response duration.

However, in a murine melanoma model, combining BRAFi, anti-PD1, and OncoVEX^mGMCSF^ (an oncolytic virus) showed enhanced efficacy in controlling tumor growth compared to single treatments. Mice receiving this triple combination had reduced tumor growth and prolonged survival. The combo increased cytotoxic T Lymphocytes (CTLs) and decreased T regulatory cells (Tregs) in tumors, favorably altering the immune microenvironment. Immunogenomic analysis revealed elevated Th1 and interferon-related genes. These findings suggest a strong rationale for combining targeted agents, oncolytic viruses, and checkpoint inhibitors for melanoma treatment, highlighting their potential synergistic effects (136). In treatment-naive melanoma, BRAF mutations shape distinct immune landscapes. BRAF-mutant melanomas exhibit fewer CD8^+^ T cells and more B cells and CD4^+^ T cells than BRAF wild-type tumors. Data from single-cell RNA sequencing, bulk RNA sequencing, flow cytometry, and immunohistochemistry validated these differences.

Interestingly, BRAF-mutant metastatic melanomas have increased CD4^+^ T cells and B cells but reduced CD8^+^ T cell infiltration versus BRAF wild-type samples. B cells in BRAF-mutant cases are potentially associated with improved survival, while Th2 cells relate to prolonged survival in BRAF wild-type cases. These findings suggest a unique immune microenvironment in BRAF-mutant melanomas that may contribute to better responses to ICI (**Figure 7B**) (137).

## 8 Future directions

### 8.1 Melanoma treatment and current limitations

Regarding melanoma treatment, three combinations of BRAF and MEK inhibitors have received approval from the FDA: vemurafenib plus cobimetinib, dabrafenib plus trametinib, and encorafenib plus binimetinib. Furthermore, combination therapy has demonstrated efficacy in treating melanoma brain metastases, although the responses have limitations (138). The FDA approved vemurafenib on August 17, 2011, based on the outcomes of the BRIM-3 trial (ClinicalTrials.gov identifier: NCT01006980), for treating patients with unresectable or metastatic BRAF^V600E^ melanoma (99). Similarly, dabrafenib received FDA approval on May 29, 2013, relying on the findings from the BREAK-3 trial (ClinicalTrials.gov identifier: NCT01227889) for the treatment of patients with unresectable or metastatic BRAF^V600E-mutated^ melanoma (**Table 1**) (111). More recently, based on the COLUMBUS trial (ClinicalTrials.gov identifier: NCT01909453), encorafenib and binimetinib secured approval for patients with unresectable or metastatic melanoma carrying BRAF^V600E/K^ mutations (72). While vemurafenib, dabrafenib, and encorafenib all belong to the class of BRAF inhibitors used to treat BRAF-mutated melanoma, the selection among them may hinge on various factors, including the patient’s specific mutation, treatment history, and individual tolerance to side effects. Combination therapies, particularly those involving MEKi like cobimetinib or trametinib, have generally exhibited superior outcomes to monotherapy and are increasingly incorporated into clinical practice. Furthermore, a comparative study was conducted to assess the cost-effectiveness of three combinations of BRAFi (encorafenib+binimetinib, cobimetinib+vemurafenib, and dabrafenib+trametinib) in managing melanoma from the perspective of a healthcare insurance provider in the United States. The findings of this study indicated that encorafenib+binimetinib emerged as the most cost-effective choice when compared to cobimetinib+vemurafenib and dabrafenib+trametinib (139).

The major limitation of BRAF/MEK-based targeted therapy is the therapeutic resistance, which can be driven by aberrant pathway activation, metabolic reprogramming, and alterations in melanoma cells’ genetic and epigenetic landscape. Vemurafenib and dabrafenib have shown significant efficacy in BRAF^V600E^-mutated melanoma. Similarly, MEKi, such as trametinib and cobimetinib, provide alternative or combinatorial therapeutic options for patients with RAS/RAF/MAP pathway-driven cancers.

**Figure 8:**
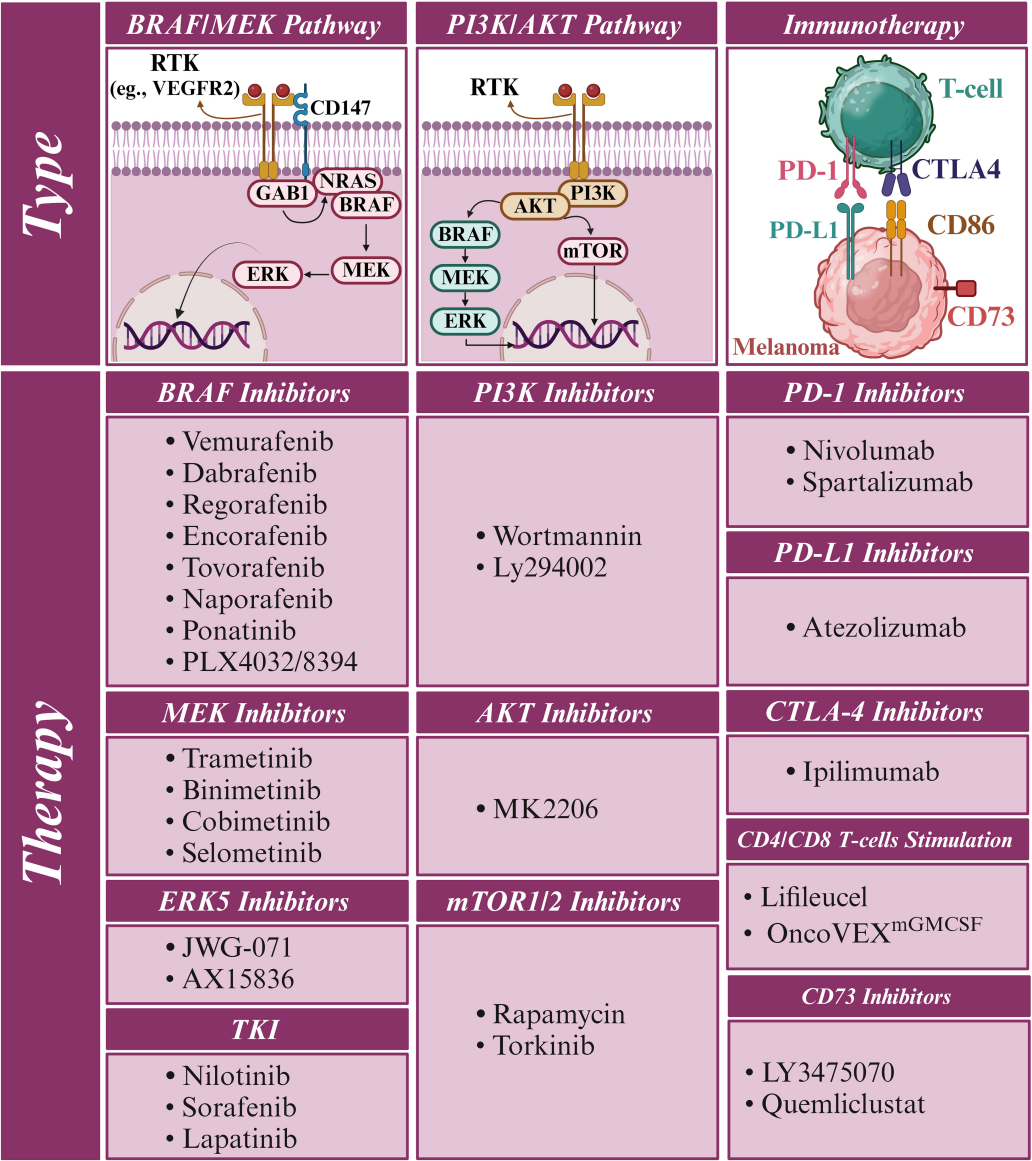
Potential druggable targets and therapeutic strategies for melanoma treatment. The schematic diagram illustrates various potential druggable targets and therapeutic approaches for the treatment of melanoma. The BRAF/MEK and PI3K/AKT signaling pathways play crucial roles in melanoma progression and are critical targets for targeted therapies. BRAF inhibitors (e.g., vemurafenib, dabrafenib), MEK inhibitors (e.g., trametinib, cobimetinib), and ERK5 inhibitors have shown significant efficacy in treating BRAF-mutated melanoma. The PI3K/AKT pathway can also be targeted using PI3K and AKT inhibitors to further suppress melanoma growth and survival. Immunotherapy has truly revolutionized melanoma treatment by leveraging the immune system’s power. Immune checkpoint inhibitors, such as PD-1 (e.g., nivolumab), PD-L1 (e.g., atezolizumab), and CTLA-4 (e.g., ipilimumab) inhibitors significantly boost anti-tumor immune responses. By inhibiting CD73, the immunosuppressive effects of adenosine can be mitigated, leading to enhanced anti-tumor immunity. Tumor-infiltrating lymphocyte (TIL) therapy (Lifileucel) is a personalized approach involving the isolation, expansion, and reinfusion of tumor-specific T cells into melanoma patients. Oncolytic virus therapy (OncoVEX^mGMCSF^) is another innovative strategy that employs genetically modified viruses to infect and lyse melanoma cells. These viruses can also stimulate immune responses by releasing tumor antigens and promoting the activation of CD4^+^ and CD8^+^ T cells. The combination of targeted therapies, immunotherapies, and novel approaches holds excellent potential for improving outcomes in melanoma patients. By targeting multiple pathways and harnessing the immune system’s power, these strategies aim to overcome resistance, minimize side effects, and provide long-lasting remission for individuals battling this aggressive form of skin cancer.

However, BRAFi, such as vemurafenib, dabrafenib, and encorafenib, primarily used to treat BRAF-mutated cancers, particularly melanoma, have been found to have unintended off-target effects on endothelial cells, leading to significant vascular complications (140). The study highlights the crucial role of endothelial cells in maintaining vascular barrier function and how BRAFi disrupts this barrier by targeting the MAPK/ERK signaling pathway and interfering with other signaling pathways that regulate cytoskeletal dynamics and cell junction integrity. This disruption leads to increased vascular permeability, as evidenced by disruption of vital junctional proteins such as cadherin-5 (VE-cadherin) and claudin-5. The off-target effects of BRAFi on endothelial cells pose potential risks for patients, including enhanced metastatic potential of tumor cells and complications related to vascular leakage. These findings underscore the importance of monitoring vascular health in patients treated with BRAFi and the need for strategies to mitigate these side effects—developing more selective BRAFi that minimizes off-target effects that protect endothelial integrity (**Figure 8**) (140).

### 8.2 Perspective druggable targets for melanoma patients

Personalized therapy in melanoma encounters a significant challenge due to the lack of precise diagnostic markers, especially in the context of BRAF/NRAS-driven melanomagenesis (**Figure 8**). Exploration in this sphere has revealed potential candidates, namely ERK5 (14), CD73 (16), the aldehyde dehydrogenase 1 family, member A1 (ALDH1A1) (141), phosphatidylserine-specific phospholipase A1-alpha (PLA1A) (5), and dermokine (DMKN) (142), as promising avenues for addressing diagnostic hurdles while facilitating tailored therapeutic decisions.

Recently, a retrospective study investigated the efficacy and safety of regorafenib, a multitargeted kinase inhibitor, in 27 advanced melanoma patients who had previously progressed on anti-PD-1, anti-CTLA-4, and BRAFi/MEKi (12). Regorafenib was administered alone or in combination with other therapies, such as nivolumab, trametinib, binimetinib, encorafenib, and dabrafenib/trametinib. The results showed a partial response in five patients (18.5%) and stable disease in three patients (11.1%). Notably, 42.8% of BRAF^V600^ mutation-positive patients treated with regorafenib combined with BRAFi/MEKi showed a partial response, including regression of brain metastases. A patient with NRAS^Q61^ mutation responded positively to regorafenib plus MEKi, while another patient with BRAF^V600^ mutation showed a partial response to regorafenib plus anti-PD-1 therapy. The study suggests that regorafenib, especially when combined with other targeted therapies, may benefit advanced melanoma patients who have exhausted other treatments, offering hope in a challenging clinical scenario (12).

Another recent study investigated the role of the ERK5 signaling pathway in BRAF^V600E^ melanoma cells with acquired resistance to dabrafenib and vemurafenib (14). The ERK5 pathway was activated in dabrafenib-resistant cells but not in vemurafenib-resistant cells, indicating distinct resistance mechanisms between the two drugs. Inhibiting ERK5 significantly reduced the viability of dabrafenib-resistant melanoma cells, while the effect was less in vemurafenib-resistant cells. Combining ERK5 inhibitors with MEK1/2 inhibitors led to a synergistic reduction in cell proliferation in dabrafenib-resistant cells but not in vemurafenib-resistant cells. The study provided insights into the molecular mechanisms underlying resistance, showing that ERK5 signaling regulates the cell cycle and survival pathways critical for maintaining resistance to dabrafenib. Therefore, the findings suggest that targeting the ERK5 pathway could be a promising therapeutic strategy to overcome acquired resistance to dabrafenib in BRAF^V600E^ melanoma (14). The research highlights the importance of understanding the distinct pathways involved in drug resistance to develop more effective treatments for melanoma. It emphasizes the potential of personalized therapeutic approaches based on specific patient resistance mechanisms.

Furthermore, a recent study has also shed light on the significant role of CD73 in melanoma’s resistance to BRAFi (**Figure 8**) (16). CD73, an enzyme involved in the production of immunosuppressive adenosine, contributed to the creation of an immunosuppressive tumor microenvironment and promoted the survival of melanoma cells under the stress of BRAFi treatment. These findings suggest CD73 could be a promising therapeutic target for overcoming resistance to BRAFi in melanoma patients. The study proposes that future treatment strategies could involve the combination of BRAFi with CD73 inhibitors to manage resistance and improve patient outcomes effectively (16).

Another recent study investigated the role of ALDH1A1, a marker indicating stem cell-like properties, in the drug resistance of melanoma cells to BRAFi/MEKi (141). The study found melanoma cells overexpressing ALDH1A1 exhibit resistance to vemurafenib and trametinib. This resistance is mediated by activating the PI3K/AKT signaling pathway rather than the MAPK pathway, helping the melanoma cells survive and proliferate (141). The role of AKT signaling in drug resistance was extensively discussed (50, 143). Based on recent studies, targeting and inhibiting the PI3K/AKT pathway can partially restore the sensitivity of melanoma cells to vemurafenib and trametinib, highlighting the significant role of this pathway in drug resistance (141, 144). Pharmacological inhibition of ALDH1A1 also leads to reduced activation of the AKT pathway and can partly restore the effectiveness of BRAFi/MEKi in treating melanoma cells. These findings propose ALDH1A1 as a potential therapeutic target, suggesting that inhibiting ALDH1A1 may help overcome resistance to BRAFi/MEKi and improve treatment outcomes for patients with melanoma.

Moreover, a comprehensive investigation positions PLA1A as a robust diagnostic marker for melanoma. Remarkably, PLA1A levels exhibit a significant elevation during melanogenesis, particularly in BRAF-mutated melanoma cases. Demonstrating impressive diagnostic performance, PLA1A effectively distinguishes BRAF-mutated melanoma samples with a sensitivity of 62% and specificity of 61% (5). This research underscores PLA1A’s potential as a prospective diagnostic tool, promising to enhance the precision of personalized therapy decisions. On the other hand, DMKN is a noteworthy marker correlated with diminished OS, especially in BRAF-mutated cases. Its modulation of the EMT-like transcriptional program positions DMKN as a multifaceted regulator (142). Collectively, DMKN and PLA1A present themselves as promising candidates for personalized therapy, shedding light on their multidimensional roles in shaping the melanoma phenotype in tailored treatment strategies.

### 8.3 Advanced therapeutic approaches

The landscape of melanoma treatment is being revolutionized by mRNA vaccines and CRISPR-Cas9, marking groundbreaking strides in personalized oncology (145, 146). mRNA vaccines intricately target genes such as BRAF and NRAS mutations to provoke robust immune responses against melanoma-associated antigens (147). Furthermore, identifying personalized neoantigens in melanomas with BRAF and NRAS mutations is pivotal in constructing immunogenic mRNA vaccines, paving the way for highly personalized melanoma therapy. Future endeavors aim to refine these vaccines for elevated efficacy while ensuring compatibility with conventional treatments, specifically addressing resistance challenges.

Regarding melanoma therapy resistance, beyond BRAF and NRAS, other pivotal genes come to the forefront. Investigations extend to genes such as PTEN, TP53, and MITF, recognized for their roles in resistance mechanisms. The ongoing research agenda encompasses exploring the broader applicability of CRISPR-Cas9 in modifying tumor microenvironments and enhancing the effectiveness of immunotherapies (146). Future projects aim to utilize CRISPR-Cas9 technology for precise targeting and modification of these genes, overcoming resistance challenges and improving the effectiveness of personalized melanoma treatment (148). This research delves deeper into broader applications, exploring changes in tumor microenvironments and innovations in immunotherapy.

Moreover, the combination of BRAF-targeted therapy with immunotherapy in the neoadjuvant setting for the treatment of melanoma was recently investigated (ClinicalTrials.gov identifier: NCT02858921) (149). The researchers found that while this combination approach can enhance immediate tumor response, it may compromise the long-term curative potential of immunotherapy. Based on these findings, researchers are against combining BRAF-targeted therapy with immunotherapy in the neoadjuvant treatment of melanoma until further follow-up data can confirm these observations. This research highlights the importance of evaluating the long-term effects of combination therapies. It emphasizes the need for continued monitoring of patient outcomes to optimize treatment strategies for melanoma.

Nevertheless, TIL therapy, particularly lifileucel (LN-44), has emerged as a promising option for metastatic melanoma, which is under review by the FDA for approval. Lifileucel, an autologous TIL therapy, has shown efficacy in recognizing specific cancer antigens. Notably, lifileucel demonstrated an ORR of 36% in patients who had failed prior anti-PD-1 treatment, with even higher response rates in patients resistant to anti-PD-1/PD-L1 treatment. Lifileucel treatment involves expanding patient-specific TIL, predominantly CD8^+^ and CD4^+^ T cells with an effector memory phenotype. These TILs can target tumor cells, migrate to tumor sites, and identify neoantigens associated with malignancies. Studies have reported long-lasting and profound responses, suggesting lifileucel as a potential therapeutic option for advanced melanoma patients with high tumor burden. Research involving 189 patients divided into two groups, with 156 receiving lifileucel TIL therapy and 25 not receiving it for various reasons, demonstrated the clinical relevance of lifileucel in heavily pre-treated advanced melanoma patients. While these results are promising, additional research is necessary to comprehend the potential of lifileucel therapy fully (**Figure 8**) (144, 145).

### 8.4 Concluding remarks

In conclusion, the review paper highlights the current landscape of BRAF-advanced therapies in melanoma, focusing on the efficacy and limitations of existing treatments such as BRAF and MEK inhibitors. The review discusses the FDA-approved combinations of inhibitors, their impact on treating BRAF-mutated melanoma, and the challenges posed by therapeutic resistance. It also delves into the off-target effects of BRAF inhibitors on endothelial cells and the importance of developing more selective therapies to minimize these effects. Moreover, the paper explores potential druggable targets for melanoma patients, including ERK5, CD73, ALDH1A1, PLA1A, and DMKN, which hold promise in addressing diagnostic hurdles and guiding personalized therapeutic decisions. Recent studies on regorafenib, ERK5 signaling, and CD73 inhibition shed light on novel strategies to overcome resistance and improve treatment outcomes in advanced melanoma cases.

Furthermore, the review touches upon advanced therapeutic tools like mRNA vaccines and CRISPR-Cas9, which are revolutionizing the field of personalized oncology by targeting specific genetic mutations and enhancing immune responses against melanoma. These cutting-edge technologies offer new avenues for tailored treatment strategies and hold great potential in improving outcomes for melanoma patients. The ongoing synergy between advancing research, targeted interventions, strategic treatment combinations, and cost-effectiveness evaluations offers a promising pathway to elevate patient outcomes significantly. This comprehensive approach stands as a beacon of hope in the persistent battle against melanoma, enabling the development of tailored and highly effective therapies for individuals facing this formidable disease.

While RAF-targeted therapy has shown remarkable efficacy in treating melanoma patients with BRAF mutations, several critical gaps and challenges persist in its clinical application. The emergence of resistance mechanisms poses a significant obstacle, as tumors can adapt to bypass RAF inhibition over time, ultimately leading to treatment failure and disease progression. Furthermore, the variability in treatment response among patients, alongside the notable toxicity profile associated with RAF inhibitors, underscores the importance of refining patient selection criteria and implementing effective management strategies to optimize treatment outcomes and quality of life. Additionally, the limited efficacy of RAF inhibitors in melanomas lacking BRAF mutations highlights the urgent need to develop alternative therapeutic approaches for this subgroup of patients. Exploring innovative combination therapies and elucidating predictive biomarkers are vital areas of ongoing research aimed at enhancing the efficacy and durability of response to RAF-targeted treatment in melanoma. Addressing these gaps through continued research efforts and clinical innovation is essential for advancing the field of melanoma treatment and ultimately improving patient outcomes and survival rates.

## References

[1] Bray F Laversanne M Sung H Ferlay J Siegel RL Soerjomataram I. Global cancer statistics 2022: GLOBOCAN estimates of incidence and mortality worldwide for 36 cancers in 185 countries. CA Cancer J Clin. (2024) 74:229–63. doi: 10.3322/caac.21834 38572751

[2] Rubinstein JC Sznol M Pavlick AC Ariyan S Cheng E Bacchiocchi A. Incidence of the V600K mutation among melanoma patients with BRAF mutations, and potential therapeutic response to the specific BRAF inhibitor PLX4032. J Transl Med. (2010) 8:67. doi: 10.1186/1479-5876-8-67 20630094 PMC2917408

[3] Czarnecka AM Bartnik E Fiedorowicz M Rutkowski P. Targeted therapy in melanoma and mechanisms of resistance. Int J Mol Sci. (2020) 21:4576. doi: 10.3390/ijms21134576 32605090 PMC7369697

[4] Morris EJ Jha S Restaino CR Dayananth P Zhu H Cooper A. Discovery of a novel ERK inhibitor with activity in models of acquired resistance to BRAF and MEK inhibitors. Cancer Discov. (2013) 3:742–50. doi: 10.1158/2159-8290.CD-13-0070 23614898

[5] Yang G Liu S Maghsoudloo M Shasaltaneh MD Kaboli PJ Zhang C. PLA1A expression as a diagnostic marker of BRAF-mutant metastasis in melanoma cancer. Sci Rep. (2021) 11:6056. doi: 10.1038/s41598-021-85595-7 33723350 PMC7961027

[6] Jabbarzadeh Kaboli P Ismail P Ling K-H. Molecular modeling, dynamics simulations, and binding efficiency of berberine derivatives: A new group of RAF inhibitors for cancer treatment. PloS One. (2018) 13:e0193941. doi: 10.1371/journal.pone.0193941 29565994 PMC5863970

[7] Vin H Ojeda SS Ching G Leung ML Chitsazzadeh V Dwyer DW. BRAF inhibitors suppress apoptosis through off-target inhibition of JNK signaling. Elife. (2013) 2:e00969. doi: 10.7554/eLife.00969 24192036 PMC3814616

[8] Bloem M van Not OJ Aarts MJB van den Berkmortel FWPJ Blank CU Blokx WAM. Adjuvant treatment with anti-PD-1 in acral melanoma: A nationwide study. Int J Cancer. (2024) 155:1455–65. doi: 10.1002/ijc.35060 38922879

[9] Tiersma JF Evers B Bakker BM Reijngoud DJ de Bruyn M de Jong S. Targeting tumour metabolism in melanoma to enhance response to immune checkpoint inhibition: A balancing act. Cancer Treat Rev. (2024) 129:102802. doi: 10.1016/j.ctrv.2024.102802 39029155

[10] Jansen P Galetzka W Lodde GC Standl F Zaremba A Herbst R. Shortened progression free and overall survival to immune-checkpoint inhibitors in BRAF-, RAS- and NF1- (“Triple”) wild type melanomas. Eur J Cancer. (2024) 208:114208. doi: 10.1016/j.ejca.2024.114208 39018633

[11] Sarnaik AA Hamid O Khushalani NI Lewis KD Medina T Kluger HM. Lifileucel, a tumor-infiltrating lymphocyte therapy, in metastatic melanoma. J Clin Oncol. (2021) 39:2656–66. doi: 10.1200/JCO.21.00612 PMC8376325 33979178

[12] Vander Mijnsbrugge A-S Cerckel J Dirven I Tijtgat J Vounckx M Claes N. Regorafenib in patients with pretreated advanced melanoma: a single-center case series. Melanoma Res. (2024) 34:366–75. doi: 10.1097/CMR.0000000000000977 38801446

[13] Chesney J Lewis KD Kluger H Hamid O Whitman E Thomas S. Efficacy and safety of lifileucel, a one-time autologous tumor-infiltrating lymphocyte (TIL) cell therapy, in patients with advanced melanoma after progression on immune checkpoint inhibitors and targeted therapies: pooled analysis of consecutive cohorts. J Immunother Cancer. (2022) 10:e005755. doi: 10.1136/jitc-2022-005755 36600653 PMC9748991

[14] Mondru AK Wilkinson B Aljasir MA Alrumayh A Greaves G Emmett M. The ERK5 pathway in BRAFV600E melanoma cells plays a role in development of acquired resistance to dabrafenib but not vemurafenib. FEBS Lett. (2024) 598:2011–27. doi: 10.1002/1873-3468.14960 38977937

[15] Espinosa-Gil S Ivanova S Alari-Pahissa E Denizli M Villafranca-Magdalena B Viñas-Casas M. MAP kinase ERK5 modulates cancer cell sensitivity to extrinsic apoptosis induced by death-receptor agonists. Cell Death Dis. (2023) 14:715. doi: 10.1038/s41419-023-06229-6 37919293 PMC10622508

[16] Giraulo C Orlando L Morretta E Voli A Plaitano P Cicala C. High levels of soluble CD73 unveil resistance to BRAF inhibitors in melanoma cells. Biomed Pharmacother. (2024) 177:117033. doi: 10.1016/j.biopha.2024.117033 38941889

[17] Ray LB Sturgill TW. Characterization of insulin-stimulated microtubule-associated protein kinase. Rapid isolation and stabilization of a novel serine/threonine kinase from 3T3-L1 cells. J Biol Chem. (1988) 263:12721–7. doi: 10.1016/S0021-9258(18)37813-X 2842341

[18] Ahn NG Seger R Bratlien RL Diltz CD Tonks NK Krebs EG. Multiple components in an epidermal growth factor-stimulated protein kinase cascade. *In vitro* activation of a myelin basic protein/microtubule-associated protein 2 kinase. J Biol Chem. (1991) 266:4220–7. doi: 10.1016/S0021-9258(20)64310-1 1705548

[19] Boulton TG Nye SH Robbins DJ Ip NY Radziejewska E Morgenbesser SD. ERKs: a family of protein-serine/threonine kinases that are activated and tyrosine phosphorylated in response to insulin and NGF. Cell. (1991) 65:663–75. doi: 10.1016/0092-8674(91)90098-j 2032290

[20] Burotto M Chiou VL Lee J-M Kohn EC. The MAPK pathway across different Malignancies: a new perspective. Cancer. (2014) 120:3446–56. doi: 10.1002/cncr.28864 PMC4221543 24948110

[21] Roskoski RJ. RAF protein-serine/threonine kinases: structure and regulation. Biochem Biophys Res Commun. (2010) 399:313–7. doi: 10.1016/j.bbrc.2010.07.092 20674547

[22] Kieser A Seitz T Adler HS Coffer P Kremmer E Crespo P. Protein kinase C-zeta reverts v-raf transformation of NIH-3T3 cells. Genes Dev. (1996) 10:1455–66. doi: 10.1101/gad.10.12.1455 8666230

[23] Sullivan RJ Infante JR Janku F Wong DJL Sosman JA Keedy V. First-in-class ERK1/2 inhibitor ulixertinib (BVD-523) in patients with MAPK mutant advanced solid tumors: results of a phase I dose-escalation and expansion study. Cancer Discov. (2018) 8:184–95. doi: 10.1158/2159-8290.CD-17-1119 29247021

[24] Chong H Vikis HG Guan K-L. Mechanisms of regulating the Raf kinase family. Cell Signal. (2003) 15:463–9. doi: 10.1016/s0898-6568(02)00139-0 12639709

[25] Lake D Corrêa SAL Müller J. Negative feedback regulation of the ERK1/2 MAPK pathway. Cell Mol Life Sci. (2016) 73:4397–413. doi: 10.1007/s00018-016-2297-8 PMC5075022 27342992

[26] Gual P Giordano S Anguissola S Parker PJ Comoglio PM. Gab1 phosphorylation: a novel mechanism for negative regulation of HGF receptor signaling. Oncogene. (2001) 20:156–66. doi: 10.1038/sj.onc.1204047 11313945

[27] Lemmon MA Schlessinger J. Cell signaling by receptor tyrosine kinases. Cell. (2010) 141:1117–34. doi: 10.1016/j.cell.2010.06.011 PMC2914105 20602996

[28] Landras A Reger de Moura C Villoutreix BO Battistella M Sadoux A Dumaz N. Novel treatment strategy for NRAS-mutated melanoma through a selective inhibitor of CD147/VEGFR-2 interaction. Oncogene. (2022) 41:2254–64. doi: 10.1038/s41388-022-02244-7 35217792

[29] Ullah R Yin Q Snell AH Wan L. RAF-MEK-ERK pathway in cancer evolution and treatment. Semin Cancer Biol. (2022) 85:123–54. doi: 10.1016/j.semcancer.2021.05.010 33992782

[30] Jabbarzadeh Kaboli P Leong MP-Y Ismail P Ling K-H. Antitumor effects of berberine against EGFR, ERK1/2, P38 and AKT in MDA-MB231 and MCF-7 breast cancer cells using molecular modelling and *in vitro* study. Pharmacol Rep. (2019) 71:13–23. doi: 10.1016/j.pharep.2018.07.005 30343043

[31] Garnett MJ Rana S Paterson H Barford D Marais R. Wild-type and mutant B-RAF activate C-RAF through distinct mechanisms involving heterodimerization. Mol Cell. (2005) 20:963–9. doi: 10.1016/j.molcel.2005.10.022 16364920

[32] Roskoski R. MEK1/2 dual-specificity protein kinases: Structure and regulation. Biochem Biophys Res Commun. (2012) 417:5–10. doi: 10.1016/j.bbrc.2011.11.145 22177953

[33] Hanks SK Quinn AM Hunter T. The protein kinase family: conserved features and deduced phylogeny of the catalytic domains. Science. (1988) 241:42–52. doi: 10.1126/science.3291115 3291115

[34] An L Jia W Yu Y Zou N Liang L Zhao Y. Lys63-linked polyubiquitination of BRAF at lysine 578 is required for BRAF-mediated signaling. Sci Rep. (2013) 3:2344. doi: 10.1038/srep02344 23907581 PMC3731650

[35] Haling JR Sudhamsu J Yen I Sideris S Sandoval W Phung W. Structure of the BRAF-MEK complex reveals a kinase activity independent role for BRAF in MAPK signaling. Cancer Cell. (2014) 26:402–13. doi: 10.1016/j.ccr.2014.07.007 25155755

[36] Gonzalez-Del Pino GL Li K Park E Schmoker AM Ha BH Eck MJ. Allosteric MEK inhibitors act on BRAF/MEK complexes to block MEK activation. Proc Natl Acad Sci USA. (2021) 118:e2107207118. doi: 10.1073/pnas.2107207118 34470822 PMC8433572

[37] Fischer A Baljuls A Reinders J Nekhoroshkova E Sibilski C Metz R. Regulation of RAF activity by 14-3-3 proteins: RAF kinases associate functionally with both homo- and heterodimeric forms of 14-3-3 proteins. J Biol Chem. (2009) 284:3183–94. doi: 10.1074/jbc.M804795200 19049963

[38] Doudican NA Orlow SJ. Inhibition of the CRAF/prohibitin interaction reverses CRAF-dependent resistance to vemurafenib. Oncogene. (2017) 36:423–8. doi: 10.1038/onc.2016.214 27321184

[39] Rajalingam K Wunder C Brinkmann V Churin Y Hekman M Sievers C. Prohibitin is required for Ras-induced Raf-MEK-ERK activation and epithelial cell migration. Nat Cell Biol. (2005) 7:837–43. doi: 10.1038/ncb1283 16041367

[40] Rajalingam K Rudel T. Prohibitin”g CRAF/MAPK activation with rocaglamides. Chem Biol. (2012) 19:1077–8. doi: 10.1016/j.chembiol.2012.09.004 22999872

[41] Boned Del Río I Young LC Sari S Jones GG Ringham-Terry B Hartig N. SHOC2 complex-driven RAF dimerization selectively contributes to ERK pathway dynamics. Proc Natl Acad Sci USA. (2019) 116:13330–9. doi: 10.1073/pnas.1902658116 PMC6613145 31213532

[42] Park E Rawson S Schmoker A Kim B-W Oh S Song K. Cryo-EM structure of a RAS/RAF recruitment complex. Nat Commun. (2023) 14:4580. doi: 10.1038/s41467-023-40299-6 37516774 PMC10387098

[43] Park E Rawson S Li K Kim B-W Ficarro SB Pino GG-D. Architecture of autoinhibited and active BRAF-MEK1-14-3-3 complexes. Nature. (2019) 575:545–50. doi: 10.1038/s41586-019-1660-y PMC7014971 31581174

[44] Sun Q Wang W. Structures of BRAF-MEK1-14-3-3 sheds light on drug discovery. Signal Transduct Target Ther. (2019) 4:59. doi: 10.1038/s41392-019-0096-z 31871776 PMC6908589

[45] Van Aelst L Barr M Marcus S Polverino A Wigler M. Complex formation between RAS and RAF and other protein kinases. Proc Natl Acad Sci USA. (1993) 90:6213–7. doi: 10.1073/pnas.90.13.6213 PMC46898 8327501

[46] Guo X Cao Y He Q Chen L Wang Q Zhang J. Modulation of the RAC1/MAPK/ERK signalling pathway by farnesyl diphosphate synthase regulates granulosa cells proliferation in polycystic ovary syndrome. Hum Cell. (2024) 37:689–703. doi: 10.1007/s13577-024-01050-5 38551774

[47] Jafari M Laraqui A Baba W Benmokhtar S El Zaitouni S Ali AA. Prevalence and patterns of mutations in RAS/RAF/MEK/ERK/MAPK signaling pathway in colorectal cancer in North Africa. BMC Cancer. (2022) 22:1142. doi: 10.1186/s12885-022-10235-w 36344948 PMC9639273

[48] Jenkins LJ Luk IY Fairlie WD Lee EF Palmieri M Schoffer KL. Genotype-tailored ERK/MAPK pathway and HDAC inhibition rewires the apoptotic rheostat to trigger colorectal cancer cell death. Mol Cancer Ther. (2023) 22:52–62. doi: 10.1158/1535-7163.MCT-22-0101 36343387 PMC9808369

[49] Shi A Liu L Li S Qi B. Natural products targeting the MAPK-signaling pathway in cancer: overview. J Cancer Res Clin Oncol. (2024) 150:6. doi: 10.1007/s00432-023-05572-7 38193944 PMC10776710

[50] Jabbarzadeh Kaboli P Salimian F Aghapour S Xiang S Zhao Q Li M. Akt-targeted therapy as a promising strategy to overcome drug resistance in breast cancer - A comprehensive review from chemotherapy to immunotherapy. Pharmacol Res. (2020) 156:104806. doi: 10.1016/j.phrs.2020.104806 32294525

[51] Hanna A Nixon MJ Estrada MV Sanchez V Sheng Q Opalenik SR. Combined Dusp4 and p53 loss with Dbf4 amplification drives tumorigenesis via cell cycle restriction and replication stress escape in breast cancer. Breast Cancer Res. (2022) 24:51. doi: 10.1186/s13058-022-01542-y 35850776 PMC9290202

[52] Prieto-Fernandez L de los Villaronga MA Hermida-Prado F Hijazi M Montoro-Jimenez I Pevida M. Driving role of head and neck cancer cell secretome on the invasion of stromal fibroblasts: Mechanistic insights by phosphoproteomics. Biomed Pharmacother. (2023) 158:114176. doi: 10.1016/j.biopha.2022.114176 36916400

[53] Ramos-Rodríguez C García-Arpa M Relea-Calatayud M González-López L Romero-Aguilera G. Metastatic melanoma negative for 5 melanocytic markers, complete regressed primary cutaneous melanoma, and melanoma-associated leukoderma in the same patient. Am J Dermatopathol. (2020) 42:956–60. doi: 10.1097/DAD.0000000000001774 32809978

[54] Lokhandwala PM Tseng L-H Rodriguez E Zheng G Pallavajjalla A Gocke CD. Clinical mutational profiling and categorization of BRAF mutations in melanomas using next generation sequencing. BMC Cancer. (2019) 19:665. doi: 10.1186/s12885-019-5864-1 31277584 PMC6612071

[55] Owsley J Stein MK Porter J In GK Salem M O’Day S. Prevalence of class I-III BRAF mutations among 114,662 cancer patients in a large genomic database. Exp Biol Med (Maywood). (2021) 246:31–9. doi: 10.1177/1535370220959657 PMC7797994 33019809

[56] Krebs FS Britschgi C Pradervand S Achermann R Tsantoulis P Haefliger S. Structure-based prediction of BRAF mutation classes using machine-learning approaches. Sci Rep. (2022) 12:12528. doi: 10.1038/s41598-022-16556-x 35869122 PMC9307832

[57] Dankner M Rose AAN Rajkumar S Siegel PM Watson IR. Classifying BRAF alterations in cancer: new rational therapeutic strategies for actionable mutations. Oncogene. (2018) 37:3183–99. doi: 10.1038/s41388-018-0171-x 29540830

[58] Qiu T Lu H Guo L Huang W Ling Y Shan L. Detection of BRAF mutation in Chinese tumor patients using a highly sensitive antibody immunohistochemistry assay. Sci Rep. (2015) 5:9211. doi: 10.1038/srep09211 25784606 PMC4363828

[59] Ascierto PA Kirkwood JM Grob J-J Simeone E Grimaldi AM Maio M. The role of BRAF V600 mutation in melanoma. J Transl Med. (2012) 10:85. doi: 10.1186/1479-5876-10-85 22554099 PMC3391993

[60] Ascierto PA Dréno B Larkin J Ribas A Liszkay G Maio M. 5-Year Outcomes with Cobimetinib plus Vemurafenib in BRAFV600 Mutation-Positive Advanced Melanoma: Extended Follow-up of the coBRIM Study. Clin Cancer Res. (2021) 27:5225–35. doi: 10.1158/1078-0432.CCR-21-0809 PMC9401485 34158360

[61] Menzies AM Long GV. Dabrafenib and trametinib, alone and in combination for BRAF-mutant metastatic melanoma. Clin Cancer Res. (2014) 20:2035–43. doi: 10.1158/1078-0432.CCR-13-2054 24583796

[62] SChadendorf D Dummer R Flaherty KT Robert C Arance A de Groot JWB. COLUMBUS 7-year update: A randomized, open-label, phase III trial of encorafenib plus binimetinib versus vemurafenib or encorafenib in patients with BRAF V600E/K-mutant melanoma. Eur J Cancer. (2024) 204:114073. doi: 10.1016/j.ejca.2024.114073 38723373

[63] McArthur GA Chapman PB Robert C Larkin J Haanen JB Dummer R. Safety and efficacy of vemurafenib in BRAF(V600E) and BRAF(V600K) mutation-positive melanoma (BRIM-3): extended follow-up of a phase 3, randomised, open-label study. Lancet Oncol. (2014) 15:323–32. doi: 10.1016/S1470-2045(14)70012-9 PMC4382632 24508103

[64] Grob J-J Amonkar MM Martin-Algarra S Demidov LV Goodman V Grotzinger K. Patient perception of the benefit of a BRAF inhibitor in metastatic melanoma: quality-of-life analyses of the BREAK-3 study comparing dabrafenib with dacarbazine. Ann Oncol. (2014) 25:1428–36. doi: 10.1093/annonc/mdu154 24769640

[65] Latimer NR Abrams KR Amonkar MM Stapelkamp C Swann RS. Adjusting for the confounding effects of treatment switching-the BREAK-3 trial: dabrafenib versus dacarbazine. Oncologist. (2015) 20:798–805. doi: 10.1634/theoncologist.2014-0429 26040620 PMC4492231

[66] SChadendorf D Amonkar MM Milhem M Grotzinger K Demidov LV Rutkowski P. Functional and symptom impact of trametinib versus chemotherapy in BRAF V600E advanced or metastatic melanoma: quality-of-life analyses of the METRIC study. Ann Oncol. (2014) 25:700–6. doi: 10.1093/annonc/mdt580 PMC4433512 24504441

[67] Flaherty KT Robert C Hersey P Nathan P Garbe C Milhem M. Improved survival with MEK inhibition in BRAF-mutated melanoma. N Engl J Med. (2012) 367:107–14. doi: 10.1056/NEJMoa1203421 22663011

[68] Grob JJ Amonkar MM Karaszewska B Schachter J Dummer R Mackiewicz A. Comparison of dabrafenib and trametinib combination therapy with vemurafenib monotherapy on health-related quality of life in patients with unresectable or metastatic cutaneous BRAF Val600-mutation-positive melanoma (COMBI-v): results of a phase 3, open-. Lancet Oncol. (2015) 16:1389–98. doi: 10.1016/S1470-2045(15)00087-X 26433819

[69] Long GV Stroyakovskiy D Gogas H Levchenko E de Braud F Larkin J. Dabrafenib and trametinib versus dabrafenib and placebo for Val600 BRAF-mutant melanoma: a multicentre, double-blind, phase 3 randomised controlled trial. Lancet. (2015) 386:444–51. doi: 10.1016/S0140-6736(15)60898-4 26037941

[70] Dummer R Long GV Robert C Tawbi HA Flaherty KT Ascierto PA. Randomized phase III trial evaluating spartalizumab plus dabrafenib and trametinib for BRAF V600-mutant unresectable or metastatic melanoma. J Clin Oncol. (2022) 40:1428–38. doi: 10.1200/JCO.21.01601 PMC9061149 35030011

[71] Ascierto PA McArthur GA Dréno B Atkinson V Liszkay G Di Giacomo AM. Cobimetinib combined with vemurafenib in advanced BRAF(V600)-mutant melanoma (coBRIM): updated efficacy results from a randomised, double-blind, phase 3 trial. Lancet Oncol. (2016) 17:1248–60. doi: 10.1016/S1470-2045(16)30122-X 27480103

[72] Dummer R Ascierto PA Gogas HJ Arance A Mandala M Liszkay G. Encorafenib plus binimetinib versus vemurafenib or encorafenib in patients with BRAF-mutant melanoma (COLUMBUS): a multicentre, open-label, randomised phase 3 trial. Lancet Oncol. (2018) 19:603–15. doi: 10.1016/S1470-2045(18)30142-6 29573941

[73] Dummer R Ascierto PA Gogas HJ Arance A Mandala M Liszkay G. Overall survival in patients with BRAF-mutant melanoma receiving encorafenib plus binimetinib versus vemurafenib or encorafenib (COLUMBUS): a multicentre, open-label, randomised, phase 3 trial. Lancet Oncol. (2018) 19:1315–27. doi: 10.1016/S1470-2045(18)30497-2 30219628

[74] Ascierto PA Dummer R Gogas HJ Flaherty KT Arance A Mandala M. Update on tolerability and overall survival in COLUMBUS: landmark analysis of a randomised phase 3 trial of encorafenib plus binimetinib vs vemurafenib or encorafenib in patients with BRAF V600-mutant melanoma. Eur J Cancer. (2020) 126:33–44. doi: 10.1016/j.ejca.2019.11.016 31901705

[75] Boussemart L Nelson A Wong M Ross JS Sosman J Mehnert J. Hybrid capture-based genomic profiling identifies BRAF V600 and non-V600 alterations in melanoma samples negative by prior testing. Oncologist. (2019) 24:657–63. doi: 10.1634/theoncologist.2018-0271 PMC6516121 30683711

[76] Comito F Aprile M Pagani R Siepe G Sperandi F Gruppioni E. Clinical characteristics and treatment outcomes of non-V600 E/K BRAF mutant melanoma patients: a single-institution experience. Melanoma Res. (2022) 32:477–84. doi: 10.1097/CMR.0000000000000854 36039514

[77] Killock D. DREAMseq of therapy for BRAF-mutant melanoma. Nat Rev Clin Oncol. (2023) 20:1. doi: 10.1038/s41571-022-00708-z 36352267

[78] Girod M Dalle S Mortier L Dalac S Leccia M-T Dutriaux C. Non-V600E/K BRAF mutations in metastatic melanoma: molecular description, frequency, and effectiveness of targeted therapy in a large national cohort. JCO Precis Oncol. (2022) 6:e2200075. doi: 10.1200/PO.22.00075 36356284

[79] Menzer C Menzies AM Carlino MS Reijers I Groen EJ Eigentler T. Targeted therapy in advanced melanoma with rare BRAF mutations. J Clin Oncol. (2019) 37:3142–51. doi: 10.1200/JCO.19.00489 PMC10448865 31580757

[80] Tkacik E Li K Gonzalez-Del Pino G Ha BH Vinals J Park E. Structure and RAF family kinase isoform selectivity of type II RAF inhibitors tovorafenib and naporafenib. J Biol Chem. (2023) 299:104634. doi: 10.1016/j.jbc.2023.104634 36963492 PMC10149214

[81] Zhang C Spevak W Zhang Y Burton EA Ma Y Habets G. RAF inhibitors that evade paradoxical MAPK pathway activation. Nature. (2015) 526:583–6. doi: 10.1038/nature14982 26466569

[82] Grasso M Estrada MA Ventocilla C Samanta M Maksimoska J Villanueva J. Chemically linked vemurafenib inhibitors promote an inactive BRAF(V600E) conformation. ACS Chem Biol. (2016) 11:2876–88. doi: 10.1021/acschembio.6b00529 PMC5108658 27571413

[83] Subbiah V Baik C Kirkwood JM. Clinical development of BRAF plus MEK inhibitor combinations. Trends Cancer. (2020) 6:797–810. doi: 10.1016/j.trecan.2020.05.009 32540454

[84] Poulikakos PI Persaud Y Janakiraman M Kong X Ng C Moriceau G. RAF inhibitor resistance is mediated by dimerization of aberrantly spliced BRAF(V600E). Nature. (2011) 480:387–90. doi: 10.1038/nature10662 PMC3266695 22113612

[85] Tsai J Lee JT Wang W Zhang J Cho H Mamo S. Discovery of a selective inhibitor of oncogenic B-Raf kinase with potent antimelanoma activity. Proc Natl Acad Sci USA. (2008) 105:3041–6. doi: 10.1073/pnas.0711741105 PMC2268581 18287029

[86] Rajakulendran T Sahmi M Lefrançois M Sicheri F Therrien M. A dimerization-dependent mechanism drives RAF catalytic activation. Nature. (2009) 461:542–5. doi: 10.1038/nature08314 19727074

[87] Roskoski RJ. Classification of small molecule protein kinase inhibitors based upon the structures of their drug-enzyme complexes. Pharmacol Res. (2016) 103:26–48. doi: 10.1016/j.phrs.2015.10.021 26529477

[88] Yen I Shanahan F Lee J Hong YS Shin SJ Moore AR. ARAF mutations confer resistance to the RAF inhibitor belvarafenib in melanoma. Nature. (2021) 594:418–23. doi: 10.1038/s41586-021-03515-1 33953400

[89] Hatzivassiliou G Song K Yen I Brandhuber BJ Anderson DJ Alvarado R. RAF inhibitors prime wild-type RAF to activate the MAPK pathway and enhance growth. Nature. (2010) 464:431–5. doi: 10.1038/nature08833 20130576

[90] Zhao Z Wu H Wang L Liu Y Knapp S Liu Q. Exploration of type II binding mode: A privileged approach for kinase inhibitor focused drug discovery? ACS Chem Biol. (2014) 9:1230–41. doi: 10.1021/cb500129t PMC4068218 24730530

[91] Sun Y Alberta JA Pilarz C Calligaris D Chadwick EJ Ramkissoon SH. A brain-penetrant RAF dimer antagonist for the noncanonical BRAF oncoprotein of pediatric low-grade astrocytomas. Neuro Oncol. (2017) 19:774–85. doi: 10.1093/neuonc/now261 PMC5464455 28082416

[92] Ramurthy S Taft BR Aversa RJ Barsanti PA Burger MT Lou Y. Design and discovery of N-(3-(2-(2-hydroxyethoxy)-6-morpholinopyridin-4-yl)-4-methylphenyl)-2-(trifluoromethyl)isonicotinamide, a selective, efficacious, and well-tolerated RAF inhibitor targeting RAS mutant cancers: the path to the clinic. J Med Chem. (2020) 63:2013–27. doi: 10.1021/acs.jmedchem.9b00161 31059256

[93] Lavoie H Therrien M. Regulation of RAF protein kinases in ERK signalling. Nat Rev Mol Cell Biol. (2015) 16:281–98. doi: 10.1038/nrm3979 25907612

[94] Gunderwala AY Nimbvikar AA Cope NJ Li Z Wang Z. Development of allosteric BRAF peptide inhibitors targeting the dimer interface of BRAF. ACS Chem Biol. (2019) 14:1471–80. doi: 10.1021/acschembio.9b00191 PMC6733264 31243962

[95] Cotto-Rios XM Agianian B Gitego N Zacharioudakis E Giricz O Wu Y. Inhibitors of BRAF dimers using an allosteric site. Nat Commun. (2020) 11:4370. doi: 10.1038/s41467-020-18123-2 32873792 PMC7462985

[96] Adamopoulos C Ahmed TA Tucker MR Ung PMU Xiao M Karoulia Z. Exploiting allosteric properties of RAF and MEK inhibitors to target therapy-resistant tumors driven by oncogenic BRAF signaling. Cancer Discov. (2021) 11:1716–35. doi: 10.1158/2159-8290.CD-20-1351 PMC8295204 33568355

[97] Bollag G Hirth P Tsai J Zhang J Ibrahim PN Cho H. Clinical efficacy of a RAF inhibitor needs broad target blockade in BRAF-mutant melanoma. Nature. (2010) 467:596–9. doi: 10.1038/nature09454 PMC2948082 20823850

[98] Sosman JA Kim KB Schuchter L Gonzalez R Pavlick AC Weber JS. Survival in BRAF V600-mutant advanced melanoma treated with vemurafenib. N Engl J Med. (2012) 366:707–14. doi: 10.1056/NEJMoa1112302 PMC3724515 22356324

[99] Chapman PB Hauschild A Robert C Haanen JB Ascierto P Larkin J. Improved survival with vemurafenib in melanoma with BRAF V600E mutation. N Engl J Med. (2011) 364:2507–16. doi: 10.1056/NEJMoa1103782 PMC3549296 21639808

[100] Larkin J Del Vecchio M Ascierto PA Krajsova I Schachter J Neyns B. Vemurafenib in patients with BRAF(V600) mutated metastatic melanoma: an open-label, multicentre, safety study. Lancet Oncol. (2014) 15:436–44. doi: 10.1016/S1470-2045(14)70051-8 24582505

[101] Ribas A Gonzalez R Pavlick A Hamid O Gajewski TF Daud A. Combination of vemurafenib and cobimetinib in patients with advanced BRAF(V600)-mutated melanoma: a phase 1b study. Lancet Oncol. (2014) 15:954–65. doi: 10.1016/S1470-2045(14)70301-8 25037139

[102] Larkin J Ascierto PA Dréno B Atkinson V Liszkay G Maio M. Combined vemurafenib and cobimetinib in BRAF-mutated melanoma. N Engl J Med. (2014) 371:1867–76. doi: 10.1056/NEJMoa1408868 25265494

[103] Deniger DC Kwong MLM Pasetto A Dudley ME Wunderlich JR Langhan MM. A pilot trial of the combination of vemurafenib with adoptive cell therapy in patients with metastatic melanoma. Clin Cancer Res. (2017) 23:351–62. doi: 10.1158/1078-0432.CCR-16-0906 PMC5245178 28093487

[104] Ascierto PA Minor D Ribas A Lebbe C O’Hagan A Arya N. Phase II trial (BREAK-2) of the BRAF inhibitor dabrafenib (GSK2118436) in patients with metastatic melanoma. J Clin Oncol. (2013) 31:3205–11. doi: 10.1200/JCO.2013.49.8691 23918947

[105] Long GV Stroyakovskiy D Gogas H Levchenko E de Braud F Larkin J. Combined BRAF and MEK inhibition versus BRAF inhibition alone in melanoma. N Engl J Med. (2014) 371:1877–88. doi: 10.1056/NEJMoa1406037 25265492

[106] Syeda MM Wiggins JM Corless BC Long GV Flaherty KT SChadendorf D. Circulating tumour DNA in patients with advanced melanoma treated with dabrafenib or dabrafenib plus trametinib: a clinical validation study. Lancet Oncol. (2021) 22:370–80. doi: 10.1016/S1470-2045(20)30726-9 PMC8034833 33587894

[107] Schumann K Mauch C Klespe K-C Loquai C Nikfarjam U Schlaak M. Real-world outcomes using PD-1 antibodies and BRAF + MEK inhibitors for adjuvant melanoma treatment from 39 skin cancer centers in Germany, Austria and Switzerland. J Eur Acad Dermatol Venereol. (2023) 37:894–906. doi: 10.1111/jdv.18779 36433688

[108] Trojaniello C Sparano F Cioli E Ascierto PA. Sequencing targeted and immune therapy in BRAF-mutant melanoma: lessons learned. Curr Oncol Rep. (2023) 25:623–34. doi: 10.1007/s11912-023-01402-8 PMC10164000 36995534

[109] Manzano JL Martin-Liberal J Fernández-Morales LA Benítez G Medina Martínez J Quindós M. Adjuvant dabrafenib and trametinib for patients with resected BRAF -mutated melanoma: DESCRIBE-AD real-world retrospective observational study. Melanoma Res. (2023) 33:388–97. doi: 10.1097/CMR.0000000000000888 PMC10470432 36988401

[110] Mezi S Botticelli A Scagnoli S Pomati G Fiscon G De Galitiis F. The impact of drug-drug interactions on the toxicity profile of combined treatment with BRAF and MEK inhibitors in patients with BRAF-mutated metastatic melanoma. Cancers (Basel). (2023) 15:4587. doi: 10.3390/cancers15184587 37760556 PMC10526382

[111] Ballantyne AD Garnock-Jones KP. Dabrafenib: first global approval. Drugs. (2013) 73:1367–76. doi: 10.1007/s40265-013-0095-2 23881668

[112] Gouda MA Subbiah V. Expanding the benefit: dabrafenib/trametinib as tissue-agnostic therapy for BRAF V600E-positive adult and pediatric solid tumors. Am Soc Clin Oncol Educ Book. (2023) 43:e404770. doi: 10.1200/EDBK_404770 37159870

[113] Sullivan RJ Weber J Patel S Dummer R Carlino MS Tan DSW. A phase ib/II study of the BRAF inhibitor encorafenib plus the MEK inhibitor binimetinib in patients with BRAF(V600E/K) -mutant solid tumors. Clin Cancer Res. (2020) 26:5102–12. doi: 10.1158/1078-0432.CCR-19-3550 32669376

[114] Dummer R Flaherty KT Robert C Arance A de Groot JWB Garbe C. COLUMBUS 5-year update: A randomized, open-label, phase III trial of encorafenib plus binimetinib versus vemurafenib or encorafenib in patients with BRAF V600-mutant melanoma. J Clin Oncol. (2022) 40:4178–88. doi: 10.1200/JCO.21.02659 PMC9916040 35862871

[115] Rose AAN. Encorafenib and binimetinib for the treatment of BRAF V600E/K-mutated melanoma. Drugs Today (Barc). (2019) 55:247–64. doi: 10.1358/dot.2019.55.4.2958476 31050693

[116] McArthur GA Maio M Arance A Nathan P Blank C Avril M-F. Vemurafenib in metastatic melanoma patients with brain metastases: an open-label, single-arm, phase 2, multicentre study. Ann Oncol. (2017) 28:634–41. doi: 10.1093/annonc/mdw641 27993793

[117] Robert C Grob JJ Stroyakovskiy D Karaszewska B Hauschild A Levchenko E. Five-year outcomes with dabrafenib plus trametinib in metastatic melanoma. N Engl J Med. (2019) 381:626–36. doi: 10.1056/NEJMoa1904059 31166680

[118] Aglietta M Chiarion-Sileni V Fava P Guidoboni M Depenni R Minisini A. Outcomes in patients with BRAF(V600)-mutated melanoma and brain metastases at baseline treated with dabrafenib plus trametinib. Tumori. (2023) 109:537–45. doi: 10.1177/03008916231179251 PMC10702308 37417313

[119] Kim Y-Y Park H Song T Choi K Dolton M Mao J. Belvarafenib penetrates the BBB and shows potent antitumor activity in a murine melanoma brain metastasis model. Clin Exp Metastasis. (2023) 40:137–48. doi: 10.1007/s10585-023-10198-7 36763292

[120] Menzies AM Long GV Kohn A Tawbi H Weber J Flaherty K. POLARIS: A phase 2 trial of encorafenib plus binimetinib evaluating high-dose and standard-dose regimens in patients with BRAF V600-mutant melanoma with brain metastasis. Neurooncol Adv. (2024) 6:vdae033. doi: 10.1093/noajnl/vdae033 38725995 PMC11079948

[121] Redmer T Schumann E Peters K Weidemeier ME Nowak S Schroeder HWS. MET receptor serves as a promising target in melanoma brain metastases. Acta Neuropathol. (2024) 147:44. doi: 10.1007/s00401-024-02694-1 38386085 PMC10884227

[122] Mahoney KM Freeman GJ McDermott DF. The next immune-checkpoint inhibitors: PD-1/PD-L1 blockade in melanoma. Clin Ther. (2015) 37:764–82. doi: 10.1016/j.clinthera.2015.02.018 PMC4497957 25823918

[123] Ascierto PA Mandalà M Ferrucci PF Guidoboni M Rutkowski P Ferraresi V. Sequencing of ipilimumab plus nivolumab and encorafenib plus binimetinib for untreated BRAF-mutated metastatic melanoma (SECOMBIT): A randomized, three-arm, open-label phase II trial. J Clin Oncol. (2023) 41:212–21. doi: 10.1200/JCO.21.02961 36049147

[124] Livingstone E Gogas H Kandolf-Sekulovic L Meier F Eigentler TK Ziemer M. Early switch from run-in treatment with vemurafenib plus cobimetinib to atezolizumab after 3 months leads to rapid loss of tumour control in patients with advanced BRAFV600-positive melanoma: The ImmunoCobiVem phase 2 randomised trial. Eur J Cancer. (2023) 190:112941. doi: 10.1016/j.ejca.2023.112941 37482012

[125] Ascierto PA Stroyakovskiy D Gogas H Robert C Lewis K Protsenko S. Overall survival with first-line atezolizumab in combination with vemurafenib and cobimetinib in BRAF(V600) mutation-positive advanced melanoma (IMspire150): second interim analysis of a multicentre, randomised, phase 3 study. Lancet Oncol. (2023) 24:33–44. doi: 10.1016/S1470-2045(22)00687-8 36460017

[126] Placzke J Rosińska M Sobczuk P Ziętek M Kempa-Kamińska N Cybulska-Stopa B. Modern approach to melanoma adjuvant treatment with anti-PD1 immune check point inhibitors or BRAF/MEK targeted therapy: multicenter real-world report. Cancers (Basel). (2023) 15:4384. doi: 10.3390/cancers15174384 PMC10486524 37686659

[127] Rozeman EA Versluis JM Sikorska K Hoefsmit EP Dimitriadis P Rao D. IMPemBra: a phase 2 study comparing pembrolizumab with intermittent/short-term dual MAPK pathway inhibition plus pembrolizumab in patients with melanoma harboring the BRAFV600 mutation. J Immunother Cancer. (2023) 11:e006821. doi: 10.1136/jitc-2023-006821 37479483 PMC10364170

[128] Dimitriou F Urner-Bloch U Eggenschwiler C Mitsakakis N Mangana J Dummer R. The association between immune checkpoint or BRAF/MEK inhibitor therapy and uveitis in patients with advanced cutaneous melanoma. Eur J Cancer. (2021) 144:215–23. doi: 10.1016/j.ejca.2020.11.027 33373866

[129] Mantia CM Werner L Stwalley B Ritchings C Tarhini AA Atkins MB. Sensitivity of treatment-free survival to subgroup analyses in patients with advanced melanoma treated with immune checkpoint inhibitors. Melanoma Res. (2022) 32:35–44. doi: 10.1097/CMR.0000000000000793 34855329 PMC8691370

[130] Kahn AM Perry CJ Etts K Kluger H Sznol M. Clinical predictors of survival in patients with BRAFV600-mutated metastatic melanoma treated with combined BRAF and MEK inhibitors after immune checkpoint inhibitors. Oncologist. (2023) 29:e507–13. doi: 10.1093/oncolo/oyad300 PMC10994263 37971411

[131] Kartolo A Yeung C Kuksis M Hopman W Baetz T. Improved overall survival in dual compared to single immune checkpoint inhibitors in BRAF V600-negative advanced melanoma. Melanoma Manag. (2022) 9:MMT60. doi: 10.2217/mmt-2021-0005 35497071 PMC9043874

[132] Zaremba A Mohr P Gutzmer R Meier F Pföhler C Weichenthal M. Immune checkpoint inhibition in patients with NRAS mutated and NRAS wild type melanoma: a multicenter Dermatologic Cooperative Oncology Group study on 637 patients from the prospective skin cancer registry ADOREG. Eur J Cancer. (2023) 188:140–51. doi: 10.1016/j.ejca.2023.04.008 37245442

[133] van Not OJ Blokx WAM van den Eertwegh AJM de Meza MM Haanen JB Blank CU. BRAF and NRAS mutation status and response to checkpoint inhibition in advanced melanoma. JCO Precis Oncol. (2022) 6:e2200018. doi: 10.1200/PO.22.00018 36130145

[134] Maeda T Yanagi T Ujiie H. Lessons from clinical trials on triple combination of immune checkpoint inhibitors and BRAF/MEK inhibitors in BRAF-mutant melanoma. Ann Transl Med. (2023) 11:326. doi: 10.21037/atm-23-1215 37405002 PMC10316103

[135] Atkins MB Lee SJ Chmielowski B Tarhini AA Cohen GI Truong T-G. Combination dabrafenib and trametinib versus combination nivolumab and ipilimumab for patients with advanced BRAF-mutant melanoma: the DREAMseq trial-ECOG-ACRIN EA6134. J Clin Oncol. (2023) 41:186–97. doi: 10.1200/JCO.22.01763 PMC9839305 36166727

[136] Gartrell RD Blake Z Rizk EM Perez-Lorenzo R Weisberg SP Simoes I. Combination immunotherapy including OncoVEX(mGMCSF) creates a favorable tumor immune micro-environment in transgenic BRAF murine melanoma. Cancer Immunol Immunother. (2022) 71:1837–49. doi: 10.1007/s00262-021-03088-y PMC10991384 34999916

[137] Wang M Zadeh S Pizzolla A Thia K Gyorki DE McArthur GA. Characterization of the treatment-naive immune microenvironment in melanoma with BRAF mutation. J Immunother Cancer. (2022) 10:e004095. doi: 10.1136/jitc-2021-004095 35383113 PMC8984014

[138] Kakadia S Yarlagadda N Awad R Kundranda M Niu J Naraev B. Mechanisms of resistance to BRAF and MEK inhibitors and clinical update of US Food and Drug Administration-approved targeted therapy in advanced melanoma. Onco Targets Ther. (2018) 11:7095–107. doi: 10.2147/OTT.S182721 PMC6200076 30410366

[139] Halloush S Alkhatib NS Almutairi AR Calamia M Halawah H Obeng-Kusi M. Economic evaluation of three BRAF + MEK inhibitors for the treatment of advanced unresectable melanoma with BRAF mutation from a US payer perspective. Ann Pharmacother. (2023) 57:1016–24. doi: 10.1177/10600280221146878 36639851

[140] Bromberger S Zadorozhna Y Ressler JM Holzner S Nawrocki A Zila N. Off-targets of BRAF inhibitors disrupt endothelial signaling and vascular barrier function. Life Sci Alliance. (2024) 7:e202402671. doi: 10.26508/lsa.202402671 38839106 PMC11153892

[141] Ciccone V Simonis V Del Gaudio C Cucini C Ziche M Morbidelli L. ALDH1A1 confers resistance to RAF/MEK inhibitors in melanoma cells by maintaining stemness phenotype and activating PI3K/AKT signaling. Biochem Pharmacol. (2024) 224:116252. doi: 10.1016/j.bcp.2024.116252 38701866

[142] Ma W Wu Z Maghsoudloo M Ijaz I Dehghan Shasaltaneh M Zhang Y. Dermokine mutations contribute to epithelial-mesenchymal transition and advanced melanoma through ERK/MAPK pathways. PloS One. (2023) 18:e0285806. doi: 10.1371/journal.pone.0285806 37432950 PMC10335698

[143] Jabbarzadeh Kaboli P Luo S Chen Y Jomhori M Imani S Xiang S. Pharmacotranscriptomic profiling of resistant triple-negative breast cancer cells treated with lapatinib and berberine shows upregulation of PI3K/Akt signaling under cytotoxic stress. Gene. (2022) 816:146171. doi: 10.1016/j.gene.2021.146171 35026293

[144] Ponzone L Audrito V Landi C Moiso E Levra Levron C Ferrua S. RICTOR/mTORC2 downregulation in BRAFV600E melanoma cells promotes resistance to BRAF/MEK inhibition. Mol Cancer. (2024) 23:105. doi: 10.1186/s12943-024-02010-1 38755661 PMC11097536

[145] Ma T Chen X Wang M. Intracellular Delivery of mRNA for Cell-Selective CRISPR/Cas9 Genome Editing using Lipid Nanoparticles. Chembiochem. (2023) 24:e202200801. doi: 10.1002/cbic.202200801 36780174

[146] Akram F Haq IU Sahreen S Nasir N Naseem W Imitaz M. CRISPR/Cas9: A revolutionary genome editing tool for human cancers treatment. Technol Cancer Res Treat. (2022) 21:15330338221132078. doi: 10.1177/15330338221132078 36254536 PMC9580090

[147] Bidram M Zhao Y Shebardina NG Baldin AV Bazhin AV Ganjalikhany MR. mRNA-based cancer vaccines: A therapeutic strategy for the treatment of melanoma patients. Vaccines (Basel). (2021) 9:1060. doi: 10.3390/vaccines9101060 34696168 PMC8540049

[148] Christodoulou E Rashid M Pacini C Droop A Robertson H van Groningen T. Analysis of CRISPR-Cas9 screens identifies genetic dependencies in melanoma. Pigment Cell Melanoma Res. (2021) 34:122–31. doi: 10.1111/pcmr.12919 PMC7818247 32767816

[149] Long GV Carlino MS Au-Yeung G Spillane AJ Shannon KF Gyorki DE. Neoadjuvant pembrolizumab, dabrafenib and trametinib in BRAFV600-mutant resectable melanoma: the randomized phase 2 NeoTrio trial. Nat Med. (2024) 30:2540–8. doi: 10.1038/s41591-024-03077-5 PMC11405264 38907159

